# Co-cultivation of filamentous microorganisms in the presence of aluminum oxide microparticles

**DOI:** 10.1007/s00253-022-12087-7

**Published:** 2022-07-30

**Authors:** Tomasz Boruta, Anna Antecka

**Affiliations:** Department of Bioprocess Engineering, Faculty of Process and Environmental Engineering, Lodz University of Technology, ul. Wolczanska 213, 93-005 Lodz, Poland

**Keywords:** *Aspergillus terreus*, Co-culture, Lovastatin, Oxytetracycline, Penicillin, Laccase

## Abstract

**Abstract:**

In the present work, the approaches of submerged co-cultivation and microparticle-enhanced cultivation (MPEC) were combined and evaluated over the course of three case studies. The filamentous fungus *Aspergillus terreus* was co-cultivated with *Penicillium rubens*, *Streptomyces rimosus*, or *Cerrena unicolor* in shake flasks with or without the addition of aluminum oxide microparticles. The influence of microparticles on the production of lovastatin, penicillin G, oxytetracycline, and laccase in co-cultures was compared with the effects recorded for the corresponding monocultures. In addition, the quantitative analyses of morphological parameters, sugars consumption, and by-products formation were performed. The study demonstrated that the influence of microparticles on the production of a given molecule in mono- and co-culture may differ considerably, e.g., the biosynthesis of oxytetracycline was shown to be inhibited due to the presence of aluminum oxide in “*A. terreus* vs. *S. rimosus*” co-cultivation variants but not in *S. rimosus* monocultures. The differences were also observed regarding the morphological characteristics, e.g., the microparticles-induced changes of projected area in the co-cultures and the corresponding monocultures were not always comparable. In addition, the study showed the importance of medium composition on the outcomes of MPEC, as exemplified by lovastatin production in *A. terreus* monocultures. Finally, the co-cultures of *A. terreus* with a white-rot fungus *C. unicolor* were described here for the first time.

**Key points:**

*• Aluminum oxide affects secondary metabolites production in submerged co-cultures.*

*• Mono- and co-cultures are differently impacted by the addition of aluminum oxide.*

*• Effect of aluminum oxide on metabolites production depends on medium composition.*

**Supplementary Information:**

The online version contains supplementary material available at 10.1007/s00253-022-12087-7.

## Introduction

Filamentous microorganisms produce a variety of biotechnologically relevant products, including secondary metabolites, enzymes, and organic acids (Sauer et al. 2008; Singh et al. 2016; van der Meij et al. 2017; Wösten 2019). Among many bioprocess-related methods developed to improve the production of valuable microbial compounds, the microparticle-enhanced cultivation (MPEC) (Kaup et al. 2008) is an example of a simple and relatively inexpensive approach. Briefly, the basic idea of MPEC is to enhance the performance of production strains by affecting their morphological phenotypes through the addition of microparticles, e.g., talc or aluminum oxide (AO), to the medium. While the effectiveness of MPEC in the context of bioprocess development has been proven (Böl et al. 2021; Karahalil et al. 2019; Meyer et al. 2021; Walisko et al. 2015), the complexity of physical and chemical interactions between the microparticles and the cultivated cells is still under intensive investigation (this topic was recently reviewed by Laible et al. 2021). Since the productivity of filamentous microorganisms is known to depend on the exhibited morphological forms (i.e., dispersed mycelia, clumps or the aggregated biomass known as “pellets”) and their characteristics (i.e., size, shape and structure), controlling the morphology in submerged cultivations is of key importance in the context of developing economically feasible biomanufacturing processes (Veiter et al. 2018; Wucherpfennig et al. 2010). Whereas the MPEC approach is typically employed for the enhancement of metabolite and enzyme titers (Antecka et al. 2016b; Coban et al. 2015a,b; Coban and Demirci 2016; Driouch et al. 2010a,b; 2012; Etschmann et al. 2015; Gonciarz and Bizukojć 2014; Kaup et al. 2008; Yatmaz et al. 2016), laboratory co-cultivation of two or more microbial species aims at triggering the cryptic biosynthetic pathways that remain inactive under monoculture conditions (Bertrand et al. 2014). Microbial interactions leading to the awakened expression of silent gene clusters have been recorded in numerous studies focused on filamentous fungi and actinomycetes (reviewed by Kim et al. 2021 and Zhuang and Zhang 2021). It should be pointed out that the co-cultivation approach was shown to be effective not only in the context of broadening the biosynthetic repertoire of microbial strains but also altering the titers of secondary metabolites relative to the results recorded for conventional monocultures (Boruta et al. 2019; Luti and Mavituna 2011a,b; Schäberle et al. 2014). Surprisingly, the two aforementioned strategies of improving microbial performance, namely MPEC and submerged co-cultivation, have never been applied simultaneously in a single bioprocess run. The impact of microparticles on the performance of mono- and co-cultures has not been yet compared and, importantly, it remains unknown if the product titers in the co-cultures enriched with mineral microparticles can exceed the ones observed in the corresponding co-cultures and monocultures propagated without the addition of microparticles. To address this, the combination of MPEC with co-cultivation was suggested and critically evaluated in the present work, which was organized in the form of three case studies. The first study was designed to involve the two well-studied and biotechnologically relevant fungal producers of secondary metabolites, namely *Aspergillus terreus* and *Penicillium rubens* (formerly *Penicillium chrysogenum*). The former species is employed for the biotechnological production of lovastatin (Mulder et al. 2015), a cholesterol-lowering substance, while the latter species is widely recognized as the producer of penicillin, a beta-lactam antibiotic (Houbraken et al. 2011). Both species represent the fungal group of *Ascomycetes* and are well-characterized in terms of their growth and secondary metabolites production under submerged conditions (Boruta and Bizukojc 2016; van den Berg 2011). Apart from their highly valued medically important molecules, *A. terreus* and *P. rubens* tend to produce the major unwanted by-products ( +)-geodin and chrysogine, respectively (Askenazi et al. 2003; Hasan et al. 2019, 2021; Pohl et al. 2020). While the liquid co-culture of *A. terreus* and *P. rubens* was characterized in a recent work of Boruta et al. (2020), the “fungus vs. fungus” co-cultivation system involving the addition of mineral particles has never been tested. In the second study, the intention was to investigate the “fungus vs. bacterium” co-cultivation under the conditions of microparticles supplementation. The “*A. terreus* vs. *Streptomyces rimosus*” co-culture, which was recently described in the context of bioreactor processes (Boruta et al. 2021), was selected to be further examined in the present work. *S. rimosus* is a producer of oxytetracycline, a popular antibiotic used predominantly for acne treatment (Zouboulis and Piquero-Martin 2003). In addition to oxytetracycline, *S.* *rimosus* commonly secretes an antifungal substance known as rimocidin (Petković et al. 2006; Song et al. 2020; Zhao et al. 2019). Finally, the third study was designed as the “*Ascomycetes* vs. *Basidiomycetes*” confrontation between the two evolutionarily distant fungal species. To provide a comparative perspective for the interpretation of results, *A. terreus* was again selected for co-cultivation. In this part of the experimental work, however, the lovastatin-producing fungus was accompanied by *Cerrena unicolor*, a white-rot fungus and a potent producer of enzymes known as laccases, the copper-containing oxidases of great industrial interest (Agustin et al. 2021; Michniewicz et al. 2006; Yang et al. 2017). The “*A. terreus* vs. *C. unicolor*” co-culture has not been described in literature so far.

The aim of the present work was to evaluate the production capabilities and morphological development in the submerged co-cultures of selected filamentous fungal and bacterial species in the presence of AO microparticles.

## Materials and methods

### Strains

The following strains were used in the study: *A. terreus* ATCC 20542, *P. rubens* (formerly *Penicillium chrysogenum*) Wisconsin 54–1255 (ATCC 28089), *S. rimosus* ATCC 10970, *C. unicolor* (Bull. ex Fr.) Murr. strain 137. The strains were purchased from the American Type Culture Collection, except *C. unicolor*, which was obtained from the Department of Biochemistry, Maria Curie-Skłodowska University (Lublin, Poland).

### Composition of solid media

The medium used for the sporulation of *A. terreus* contained: malt extract 20 g L^−1^, casein peptone 5 g L^−1^, and agar 20 g L^−1^. The spores of *P. rubens* were prepared on the potato dextrose agar: 300 mg of potatoes were boiled in 500 mL of distilled water for 20 min and allowed to steep for 30 min; then, the filtrate was supplemented with glucose (20 g L^−1^) and agar (20 g L^−1^). For *S. rimosus*, the ISP2 medium (Becton Dickinson, USA) was applied. *C. unicolor* was maintained on malt extract agar (Antecka et al. 2016b; Janusz et al. 2007). The solid media were autoclaved at 121 °C for 15 min.

### Composition of liquid media

The composition of liquid medium, formulated based on previous literature, is provided below for each of the three case studies. The formulated media was used for both mono- and co-cultivation.

“*A. terreus* vs. *P. rubens*” case study: glucose 10 g L^−1^; lactose 30 g L^−1^; yeast extract 6 g L^−1^; KH_2_PO_4_ 1.51 g L^−1^; phenylacetic acid 0.5 g L^−1^; NaCl 0.4 g L^−1^; MgSO_4_·7 H_2_O 0.51 g L^−1^; ZnSO_4_·7 H_2_O 1 mg L^−1^, Fe(NO_3_)_3_·9 H_2_O 2 mg L^−1^; biotin 0.04 mg L^−1^; 1 mL L^−1^ of the following trace elements solution: MnSO_4_ 50 mg L^−1^; Na_2_B_4_O_7_·10 H_2_O 100 mg L^−1^; Na_2_MoO_4_·2 H_2_O 50 mg L^−1^; and CuSO_4_·5 H_2_O 250 mg L^−1^ (Boruta et al. 2020).

“*A. terreus* vs. *S. rimosus*” case study: glucose 20 g L^−1^; lactose 20 g L^−1^; yeast extract 5 g L^−1^; KH_2_PO_4_ 1.51 g L^−1^; NaCl 0.4 g L^−1^; MgSO_4_·7 H_2_O 0.51 g L^−1^; ZnSO_4_·7 H_2_O 1 mg L^−1^; Fe(NO_3_)_3_·9 H_2_O 2 mg L^−1^; biotin 0.04 mg L^−1^; 1 mL L^−1^ of the following trace elements solution: MnSO_4_ 50 mg L^−1^; Na_2_B_4_O_7_·10 H_2_O 100 mg L^−1^; Na_2_MoO_4_·2 H_2_O 50 mg L^−1^, and CuSO_4_·5 H_2_O 250 mg L^−1^ (Boruta et al. 2021).

“*A. terreus* vs. *C. unicolor*” case study: glucose 10 g L^−1^; L-asparagine 1.5 g L^−1^; yeast extract 2 g L^−1^; KH_2_PO_4_ 0.47 g L^−1^; MgSO_4_·7 H_2_O 0.5 g L^−1^; Na_2_HPO_4_·12 H_2_O 0.48 g L^−1^; Mn(CH_3_COO)_2_·4 H_2_O 12 mg L^−1^; Zn(NO_3_)_2_·6 H_2_O 3.14 mg L^−1^; CuSO_4_·5 H_2_O 3.19 mg L^−1^; Ca(NO_3_)_2_·4 H_2_O 50 mg L^−1^; FeCl_3_·6 H_2_O 3.2 g L^−1^; and thiamine 50 µg L^−1^ (Antecka et al. 2016b; Janusz et al. 2007).

For the microparticle-containing variants, the microparticles (mean diameter: 10 µm) of aluminum oxide (Al_2_O_3_) were added to the medium at the concentration of 6 g L^−1^. The microparticles were sterilized separately for 30 min at 121 °C as dry powder.

The pH of all the media was adjusted to 6.5 with the use of NaOH solution. The media were autoclaved for 20 min at 121 °C.

### Cultivation on solid media

The biomass formed on solid media provided the basis for the inoculation of liquid media. The solid medium for the sporulation of *A. terreus*, *P. rubens*, and *S. rimosus* was prepared in test tubes in the form of agar slants. The slants were inoculated with spore suspension (approx. 10^9^ spores per liter) and incubated for 7 days at 26 °C in a laboratory incubator. In the case of *C. unicolor*, Petri plates were employed to obtain the fungal biomass for liquid medium inoculation. The incubation of plates with *C. unicolor* was carried out at 28 °C for 14 days.

### Cultivation in liquid medium

The co-cultures were initiated by co-inoculating liquid medium with the spores of two strains together with AO microparticles. In each case, the spore suspension was obtained by transferring the spores from an agar slant with a sterile 1-mL pipette into a portion of sterile medium to reach the concentration of approx. 10^9^ spores per liter (the procedure was developed and monitored with the use of a Thoma chamber). The exception was *C. unicolor*, for which the spores were not available, and the mycelium harvested from overgrown Petri plates was homogenized (as described by Antecka et al. 2016b) and a 7.5-mL portion of the homogenized biomass suspension was used to inoculate 150 mL of the medium. The amount of *C. unicolor* mycelium harvested from the plates was adjusted to achieve the initial biomass concentration in the medium at the level of 0.5 g L^−1^. In the case of the corresponding AO monocultures, the AO microparticles were added to the medium at 6 g L^−1^ together with a single species at the time of inoculation. The non-AO mono- and co-cultures were initiated in the same manner as their AO counterparts with the exception that no microparticles were used in these cases. The cultivation was performed in a shaking incubator Certomat® BS-1 (B. Braun Biotech International, Germany) at 28 °C and the rotary speed of 110 min^−1^. Flat-bottomed shake flasks (working volume of 150 mL and the total volume of 500 mL) were used throughout the study. The time of cultivation for the “*A. terreus* vs *P. rubens*” and “*A. terreus* vs. *S. rimosus*” case studies was equal to 168 h, whereas in the “*A. terreus* vs. *C. unicolor*” case study the process was performed for 216 h. The reason for having a longer cultivation time in the third case study was that *C. unicolor* requires 216 h to display its laccase-producing capabilities (Antecka et al. 2016b).

### Chemical analysis

The biomass was removed by filtration and the filtrate was analyzed with the use of ultra-performance liquid chromatography Acquity system coupled with a high-resolution mass spectrometer Synapt G2 (Waters, USA). The concentration values and peak areas of secondary metabolites were determined with the use of TargetLynx (Waters, USA). The details of chromatographic separation and mass spectrometry-related parameters were previously provided by Bizukojć et al. (2012). The peak areas of ( +)-geodin, chrysogine, and rimocidin were determined with the use of ESI^−^ quantification traces *m/z* = 396.989, 173.069, and 766.3990, respectively. The assay of laccase activity in the culture filtrate was performed as previously described (Antecka et al. 2019).

### Morphological analysis

Microscopic images of filamentous morphology were collected with the use of Olympus BX53 light microscope (Olympus Corporation, Japan). The digital image analysis of pellets was conducted with the aid of Olympus cellSens Dimension Desktop 1.16 software (Olympus Corporation, Japan). The methodological details of morphological analysis can be found in the previous report of Kowalska et al. (2018). The microscopic images required to determine the values of morphological parameters were gathered over the course of 3 independent experiments. At least 15 filamentous objects representing a given variant (e.g., the *A. terreus* monoculture) were analyzed during each of the independent experiments. Altogether, every investigated variant was thus represented by at least 45 objects that were used to calculate the mean value and the standard deviation (SD) of the given morphological parameter value. The definitions of the analyzed morphological parameters are as follows (after Kowalska et al. 2018): projected area is the number of pixels in the given object multiplied by the squared unit of calibration; elongation is a squared quotient of longitudinal and transversal deviation of pixels in the given object along the line of regression (an ideally circular object has the elongation value equal to 1); roughness is calculated as the ratio of projected area to convex area (an ideally smooth and convex object has the roughness value equal to 1). The parameters were determined for each of the analyzed filamentous objects by using the built-in functions of Olympus cellSens Dimension Desktop 1.16 software (Olympus Corporation, Japan). Prior to parameter determination, the software-assisted and manually curated identification of the object (i.e., isolation of the object from the background) was performed.

### Statistical analysis

The data were gathered in three independent experiments. The results of all measurements were presented as the “mean value ± SD.” For the chemical analyses of target products and carbon sources, the statistical analysis (*n* = 3) was performed in OriginPro (OriginLab, USA). For the morphological analyses, the data was extracted from Olympus cellSens Dimension Desktop 1.16 software (Olympus Corporation, Japan) and processed in OriginPro (OriginLab, USA). Regarding the determination of morphological parameters, the average number of pellets considered for each datapoint was equal to 45. The two-sample *t*-test (with a significance level of *α* = 0.05) was applied to verify if the results obtained for the AO variants differed significantly from their non-AO counterparts.

## Results

The submerged liquid co-cultures of *A. terreus* with *P. rubens*, *S. rimosus*, or *C. unicolor* were evaluated with respect to their morphological characteristics, production-related performance, and the consumption of carbon sources. The quantitative assessment of pellet morphology was performed by considering the size and shape parameters calculated on the basis of microscopic images, namely the projected area, elongation, and roughness. Each of the three conducted case studies involved the comparative analysis of six experimental variants, i.e., the monoculture of *A. terreus* with and without the addition of AO, the two-species co-culture of *A. terreus* with its partner with and without AO, and, finally, the monoculture of the partner strain itself with and without AO. Over the course of the experimental work, the impact of AO addition on the performance of mono- and co-cultures was comparatively assessed.

### *A. terreus* versus *P. rubens*

**Fig. 1:**
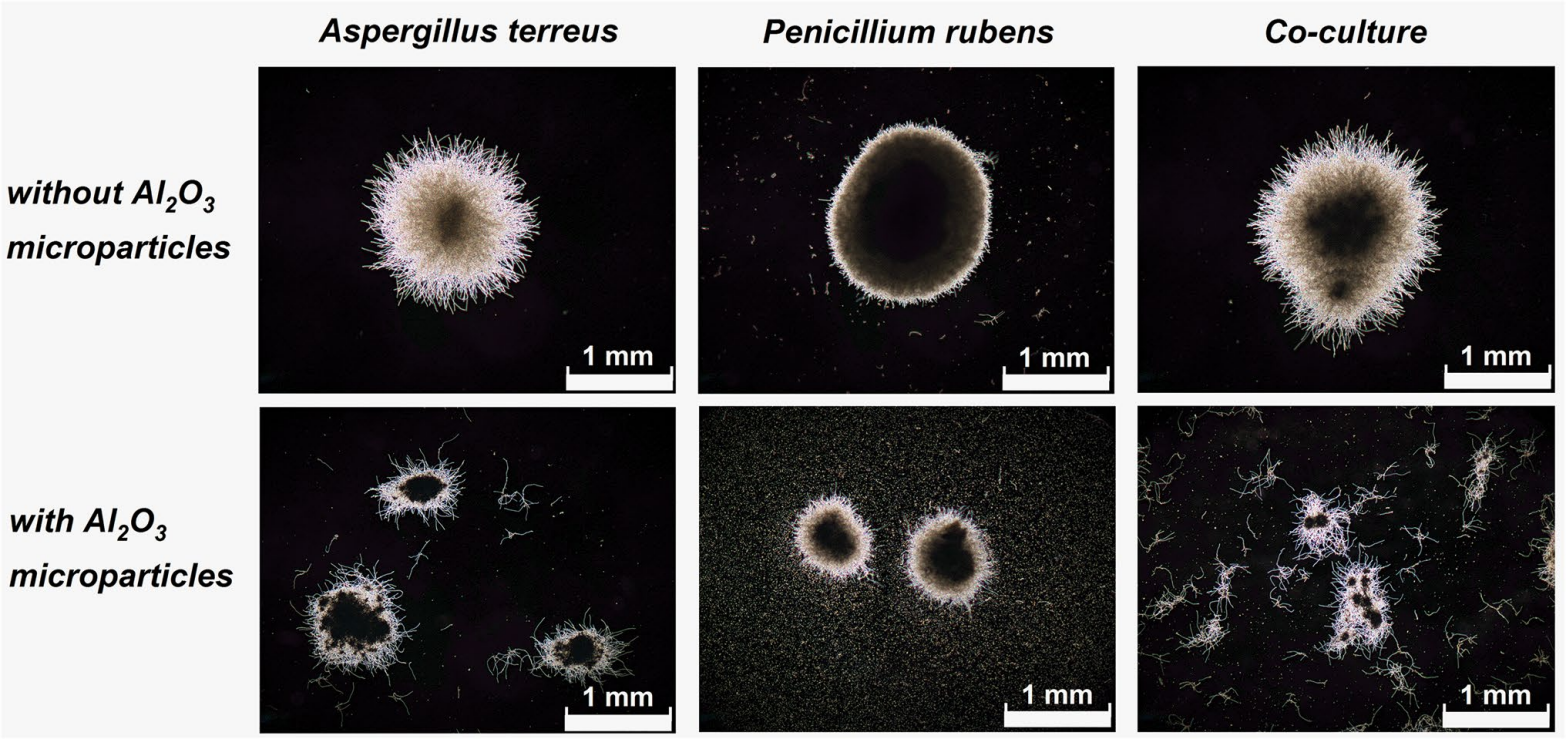
Influence of aluminum oxide (AO) on the morphology of *Aspergillus terreus* and *Penicillium rubens* grown in mono- and co-cultures. Microscopic images of *A. terreus* monoculture, *P. rubens* monoculture, and the “*A. terreus* vs. *P. rubens*” co-culture with and without the addition of AO are shown. The images were obtained after 24 h of cultivation. In all investigated variants, the supplementation with AO resulted in the decrease of pellet size compared to the non-AO counterparts. The medium used for the cultivation contained glucose (10 g L^−1^), lactose (30 g L^−1^), yeast extract (6 g L^−1^), KH_2_PO_4_ (1.51 g L^−1^), phenylacetic acid (0.5 g L^−1^), biotin (0.04 mg L^−1^), and salts. At least 45 objects were analyzed for each experimental variant, the selected examples are depicted here

**Fig. 2:**
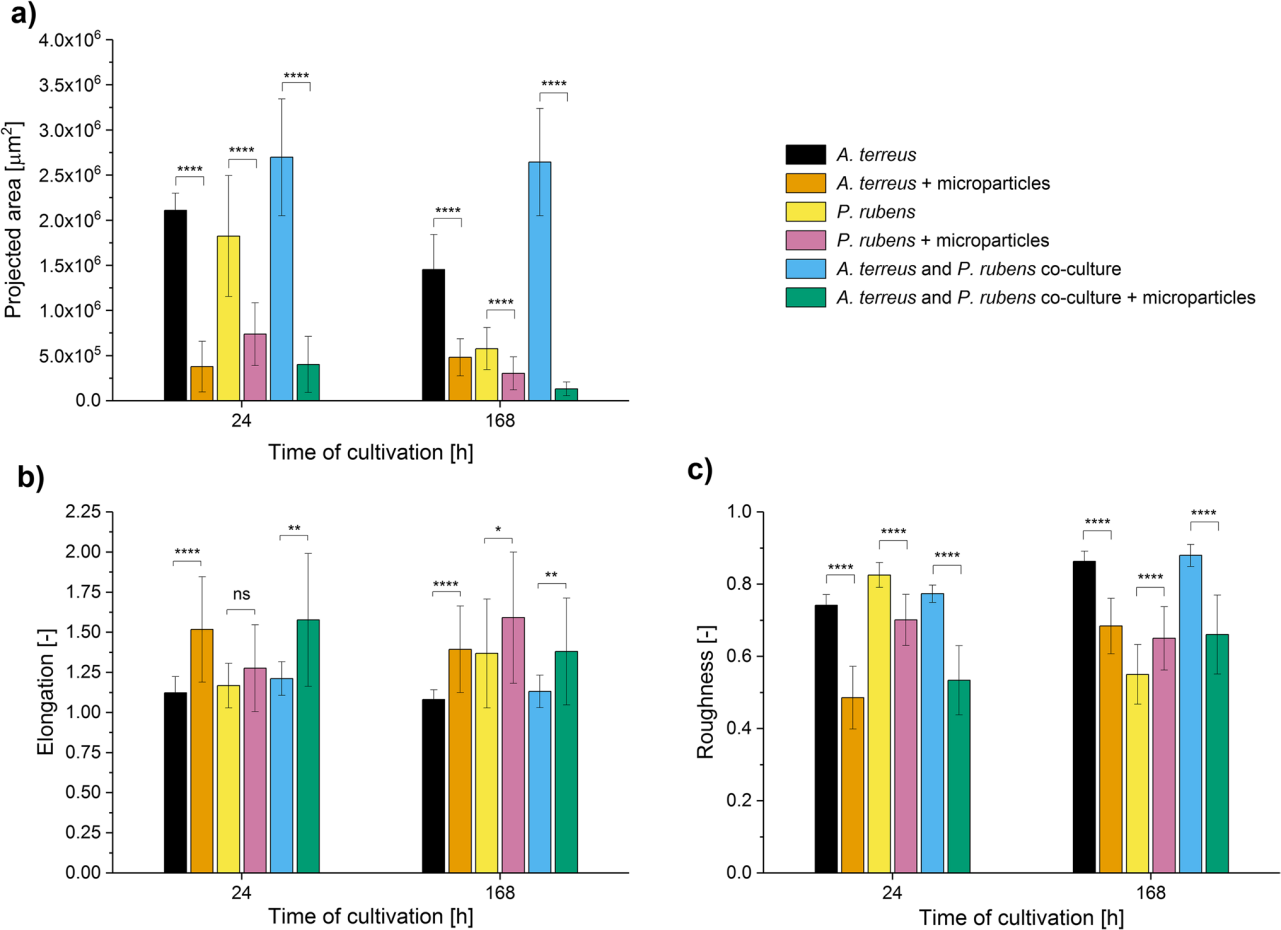
Influence of aluminum oxide (AO) on the values of morphological parameters determined for *Aspergillus terreus* and *Penicillium rubens* grown in mono- and co-cultures. Results of quantitative morphological analysis of pellets developed in the *A. terreus* monoculture, *P. rubens* monoculture and the “*A. terreus* vs. *P. rubens*” co-culture with and without the addition of AO microparticles are shown. The values of projected area (**a**), elongation (**b**), and roughness (**c**) parameters are presented as the mean value ± standard deviation with the average number of analyzed objects (*n*) equal to 45. The two-sample t-test (with a significance level of *α* = 0.05) was applied to verify if the results obtained for the AO variants differed significantly from their non-AO counterparts. *p ≤ 0.05, **p ≤ 0.01, ****p ≤ 0.0001, ns, not significant

In the confrontation between *A. terreus* and *P. rubens*, the presence of AO led to the visible morphological changes within the developing mycelial pellets (Fig. 1). In the 24 h of the run, the addition of AO to the monoculture of *A. terreus*, monoculture of *P. rubens*, and the “*A. terreus* vs. *P. rubens*” co-culture led to the 5.6-, 2.5-, and 6.7-fold decrease of mean projected area values, respectively, compared with the corresponding variants propagated without AO (Fig. 2a). In the last day of the process (168 h), however, there was a contrast between the co-cultures and the remaining datasets regarding the factor by which the pellets in the AO medium differed from their non-AO counterparts. In the case of the co-cultures, the addition of AO resulted in the 20-fold decrease of mean projected area (a difference that was greater than the one recorded in the 24 h of the run for the same pair of variants), whereas in the *A. terreus* and *P. rubens* monocultures, the 3.0- and 1.9-fold microparticle-related decrease was noted (the differences less prominent than in 24 h) (Fig. 2a). Overall, the co-culture enriched with AO exhibited the smallest mean value of the projected area among the variants tested in the “*A. terreus* vs. *P. rubens*” study. Whereas the addition of AO led to the decrease of mean projected area in all the examined culture sets (Fig. 2a), the opposite effect was observed with regard to the elongation (Fig. 2b). It should be noted here that the variability within the projected area and elongation values was rather high, as illustrated by the error bars in Fig. 2a and b, respectively. As far as the third morphological parameter was concerned, namely roughness, the presence of AO in co-cultures and *A. terreus* monocultures resulted in the decreased parameter values compared to the non-AO counterparts, while in the case of *P. rubens* monocultures, the influence of microparticles on the value of roughness changed over time. After 24 h, the mycelial pellets in the AO variant of *P. rubens* were characterized by a smaller mean value of roughness than the structures in the non-AO cultivation, but after 168 h, this trend was reversed (Fig. 2c). It was also noticed that the mean value of projected area recorded at 24 h for a given variant was smaller than the mean projected area recorded for the same variant at 168 h, with the exception of *A. terreus* AO monoculture (Fig. 2a). The same trend was observed for the mean elongation value in *A. terreus* monocultures and co-cultures but not in the case of *P. rubens* monocultivations (regardless the presence or absence of AO) (Fig. 2b). By contrast, only in the *P. rubens* monocultures did the mean roughness value decrease between 24 and 168 h (Fig. 2c).

**Fig. 3:**
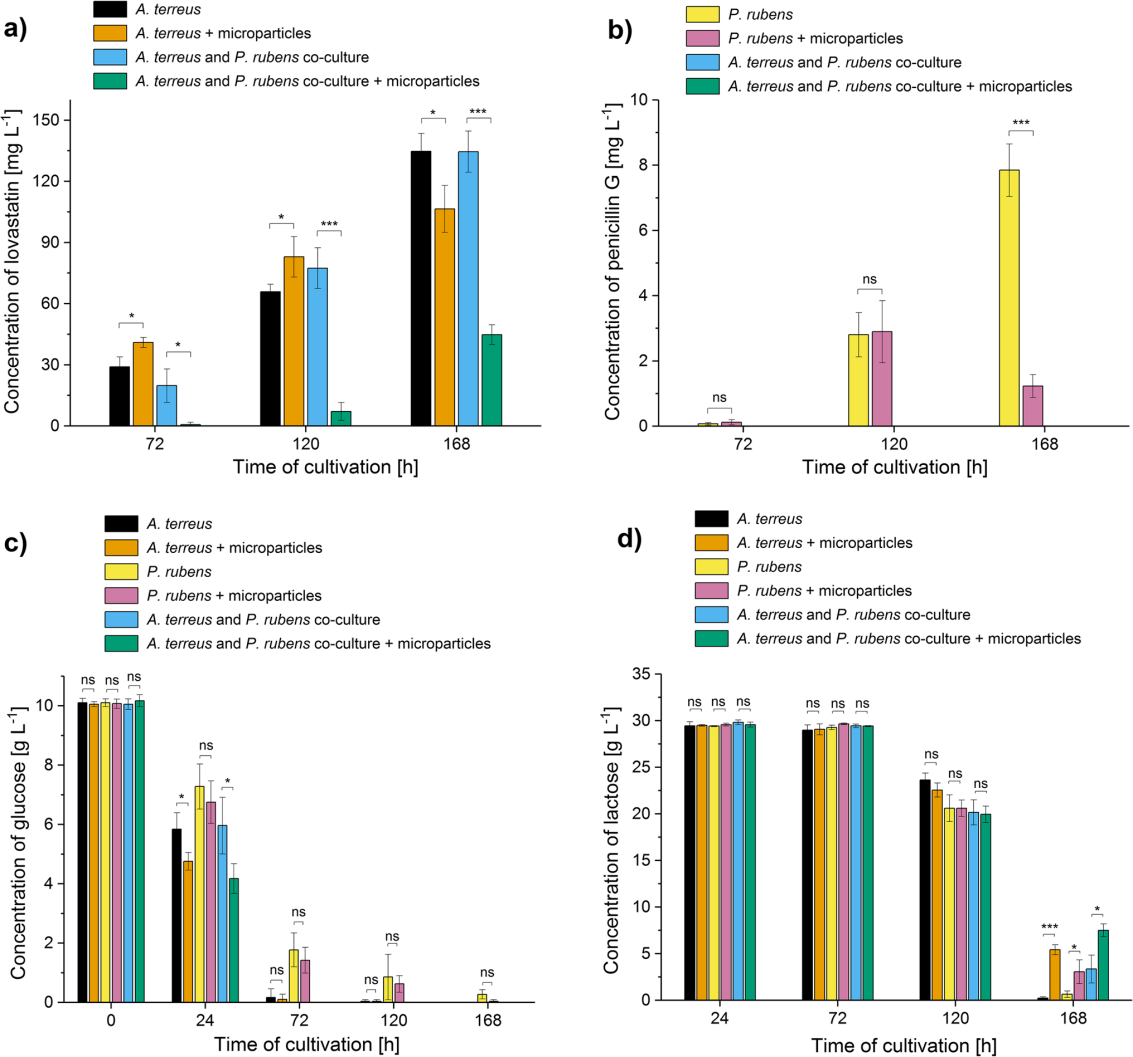
Influence of aluminum oxide (AO) on the titers of secondary metabolites and the levels of carbon substrates in the mono- and co-cultures of *Aspergillus terreus* and *Penicillium rubens*. Time courses of lovastatin (**a**), penicillin G (**b**), glucose (**c**), and lactose (**d**) levels in the *A. terreus* monoculture and/or or *P. rubens* monoculture and the “*A. terreus* vs. *P. rubens*” co-culture with and without the addition of AO microparticles are shown. The concentration values are presented as “mean ± standard deviation” (*n* = 3). No traces of lovastatin or penicillin G were detected in 24 h of the cultivation run. The production of penicillin G was confirmed solely in *P. rubens* monocultures. The two-sample t-test (with a significance level of *α* = 0.05) was applied to verify if the results obtained for the AO variants differed significantly from their non-AO counterparts. *p ≤ 0.05, ***p ≤ 0.001, ns, not significant

The production of lovastatin in *A. terreus* monocultures was found to be enhanced due to the supplementation with AO, but the stimulatory effect was clearly time-dependent, i.e., it was observed in 72 and 120 h of the run but not after 168 h (Fig. 3a). Surprisingly, the presence of AO in the co-cultures led to markedly different outcomes, i.e., the biosynthesis of lovastatin was visibly inhibited regardless of the time of the process (Fig. 3a). For example, in 120 h of the run, the AO co-cultures showed a 10.9-fold decrease in lovastatin titers compared to the co-cultivations performed without AO (Fig. 3a). Regarding the second analyzed metabolite, namely penicillin G, its production was confirmed exclusively in the monocultures of *P. rubens* (Fig. 3b). The absence of this molecule in co-cultures was recorded for both tested scenarios, i.e., for the presence and absence of AO in the broth. Interestingly, until 120 h, there were no significant differences between the AO and non-AO monocultures in terms of penicillin G levels. This contrasted with the drastic differences noted in 168 h of the run, where the presence of AO was found to be detrimental for the biosynthesis of penicillin G (Fig. 3b). Apart from lovastatin and penicillin G, the secondary metabolites ( +)-geodin and chrysogine were found to be produced by *A. terreus* and *P. rubens*, respectively. The former molecule was detected not earlier than in 120 h of the run, whereas the presence of chrysogine was noted already in 72 h (Fig. S1). In the case of ( +)-geodin production, the addition of AO clearly resulted in the inhibitory effect and the differences between the AO and the non-AO variants were evident, especially among the co-cultures (Fig. S1a). By contrast, the presence of AO led to the enhanced production of chrysogine both in the mono- and the co-cultivation set (Fig. S1b).

Regarding the consumption of carbon substrates, it was observed that the presence of AO generally stimulated the catabolism of glucose, especially in *A. terreus* monocultures and the co-cultures. After 24 h of growth, the lowest mean value of this sugar was recorded for the AO co-culture (Fig. 3c). Then, at 72 h, it was found that glucose was completely depleted in both tested co-cultivation variants (with and without AO), while the trace amounts of residual glucose could still be detected in the *A. terreus* monocultures. What is more, the consumption of this carbon substrate in the *P. rubens* monocultures was relatively slow if compared to *A. terreus* monocultures and the co-cultures. In this case, the presence of glucose was still confirmed after 168 h of the run (Fig. 3c). When the catabolism of the second carbon source (lactose) was considered, it became clear that the corresponding concentration pattern (Fig. 3d) did not resemble the one recorded for glucose. During the initial 72 h of cultivation, lactose catabolic pathways were repressed regardless the variant, and no decrease of lactose levels was observed. Later, in 120 h of the run, the consumption of lactose was no longer seen to be blocked. After 168 h of the process, the AO co-cultures were characterized by the highest residual levels of lactose (Fig. 3d). This contrasted the observations made regarding glucose, which in the case of co-cultures was depleted earlier than in the remaining variants (Fig. 3c).

### *A. terreus* versus *S. rimosus*

**Fig. 4:**
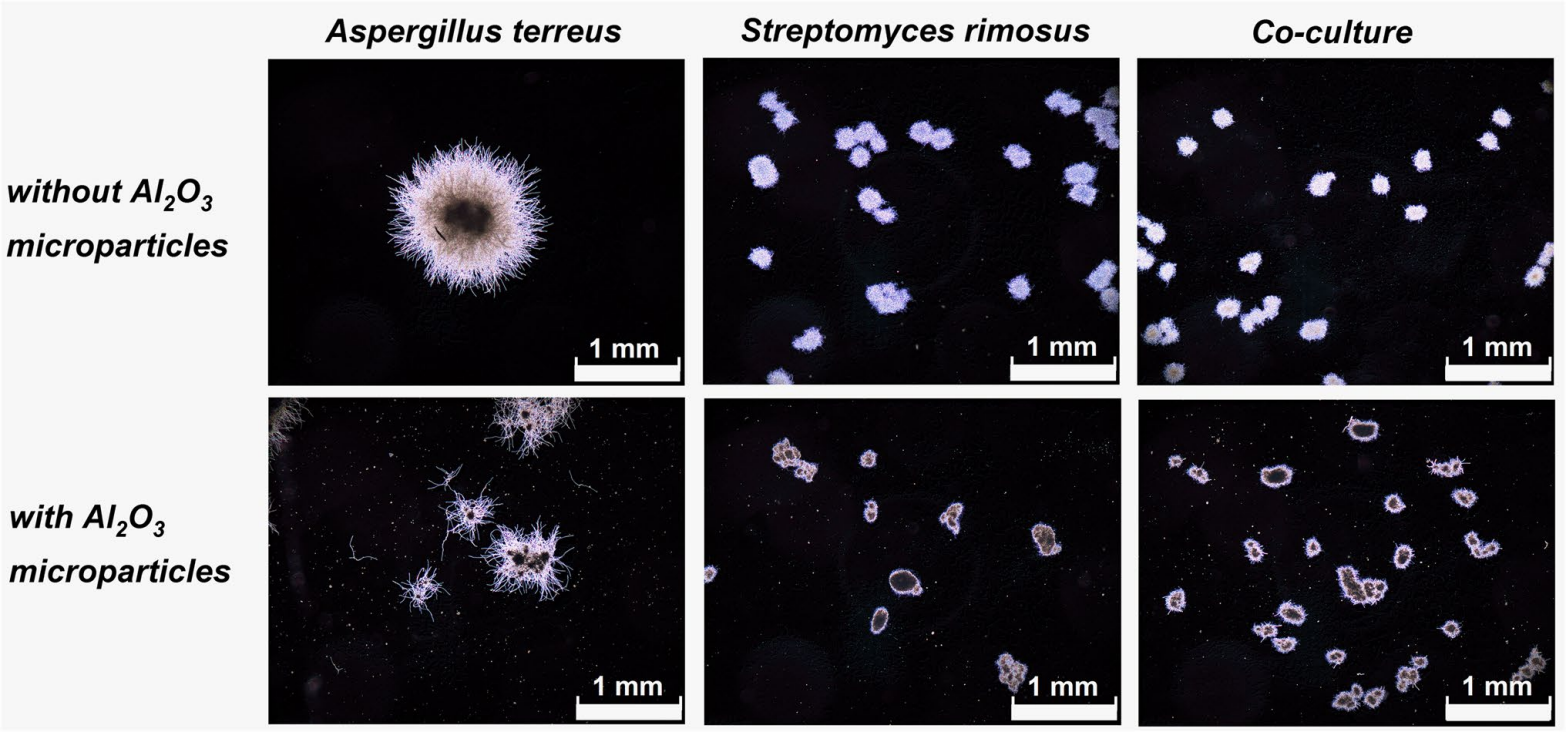
Influence of aluminum oxide (AO) on the morphology of *Aspergillus terreus* and *Streptomyces rimosus* grown in mono- and co-cultures. Microscopic images of *A.* *terreus* monoculture, *S. rimosus* monoculture and the “*A. terreus* vs. *S. rimosus*” co-culture with and without the addition of AO microparticles are shown. The images were obtained after 24 h of cultivation. The supplementation with AO resulted in the decrease of pellet size of *A. terreus* compared to the non-AO counterparts, whereas in the *S. rimosus* monocultures and the co-cultures this effect was not observed. The medium used for the cultivation contained glucose (20 g L^−1^), lactose (20 g L^−1^), yeast extract (5 g L^−1^), KH_2_PO_4_ (1.51 g L^−1^), biotin (0.04 mg L^−1^), and salts. At least 45 objects were analyzed for each experimental variant, the selected examples are depicted here

**Fig. 5:**
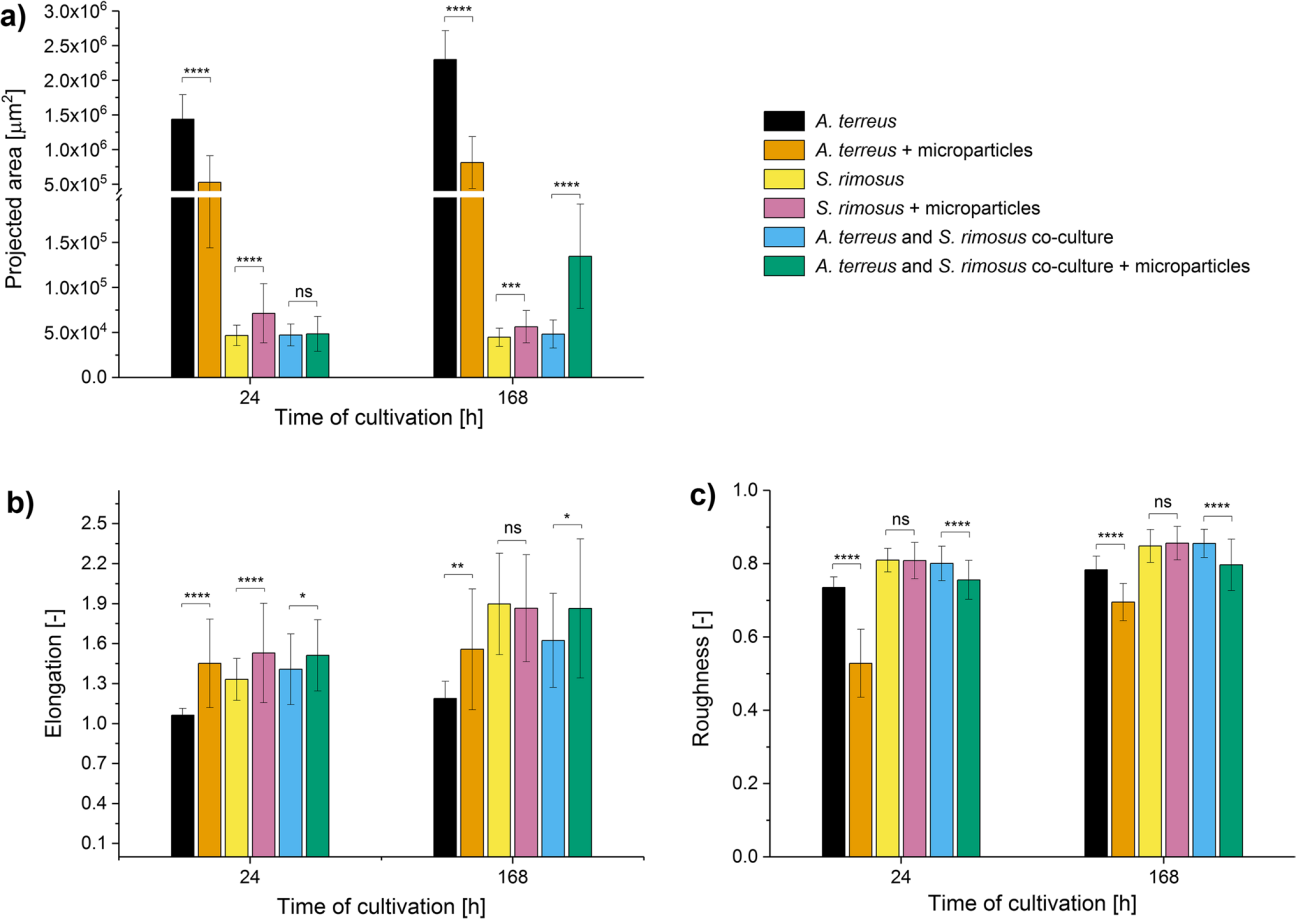
Influence of aluminum oxide (AO) on the values of morphological parameters determined for *Aspergillus terreus* and *Streptomyces rimosus* grown in mono- and co-cultures. Results of quantitative morphological analysis of pellets developed in the *A. terreus* monoculture, *S. rimosus* monoculture, and the “*A. terreus* vs. *S. rimosus*” co-culture with and without the addition of AO microparticles are shown. The values of projected area (**a**), elongation (**b**), and roughness (**c**) parameters are presented as the mean value ± standard deviation with the average number of analyzed objects (*n*) equal to 45. The two-sample *t*-test (with a significance level of *α* = 0.05) was applied to verify if the results obtained for the AO variants differed significantly from their non-AO counterparts. *p ≤ 0.05, **p ≤ 0.01, ***p ≤ 0.001, **** *p* ≤ 0.0001, ns, not significant

When the microscopic images in the “*A. terreus* vs. *S. rimosus*” case study were considered (Fig. 4), the morphological differences between the co-cultures and the *S. rimosus* monocultures were not immediately recognizable. In both cases, the presence of AO resulted in the development of pellets with a compact and dense core (Fig. 4). The results of digital image analysis revealed that at 24 h, there were no significant differences regarding the mean projected area values between the co-cultures containing AO and the ones without AO. At 168 h, however, they differed by the factor of 2.8 (Fig. 5a). The mean value of projected area in AO co-cultures increased considerably between 24 and 168 h of the run and, at the same time, exhibited the increase in variability within the tested population of objects, as indicated by the error bars in Fig. 5a. The changes in mean projected area values that occurred between 24 and 168 h were visibly smaller in AO monocultures than in AO co-cultures (Fig. 5a). At both considered time points, the addition of AO resulted in the decrease of mean projected area in the monocultures of *A. terreus* but led to the opposite effect in the monocultures of *S. rimosus* (Fig. 5a). At 24 h, it became evident that the use of AO was responsible for the increase of mean elongation value in *A. terreus* monocultures, *S. rimosus* monocultures, and the co-cultures compared to their non-AO counterparts. However, at 168 h, this observation remained valid for the monocultures of *A. terreus* and the co-cultures but not for the monocultures of *S. rimosus* (Fig. 5b). As far as the roughness parameter was concerned, the addition of AO led to the visible decrease of mean values in *A. terreus* monocultures and the co-cultures but not in *S. rimosus* monocultures (Fig. 5c). This behavior was observed at 24 and 168 h of the run.

**Fig. 6:**
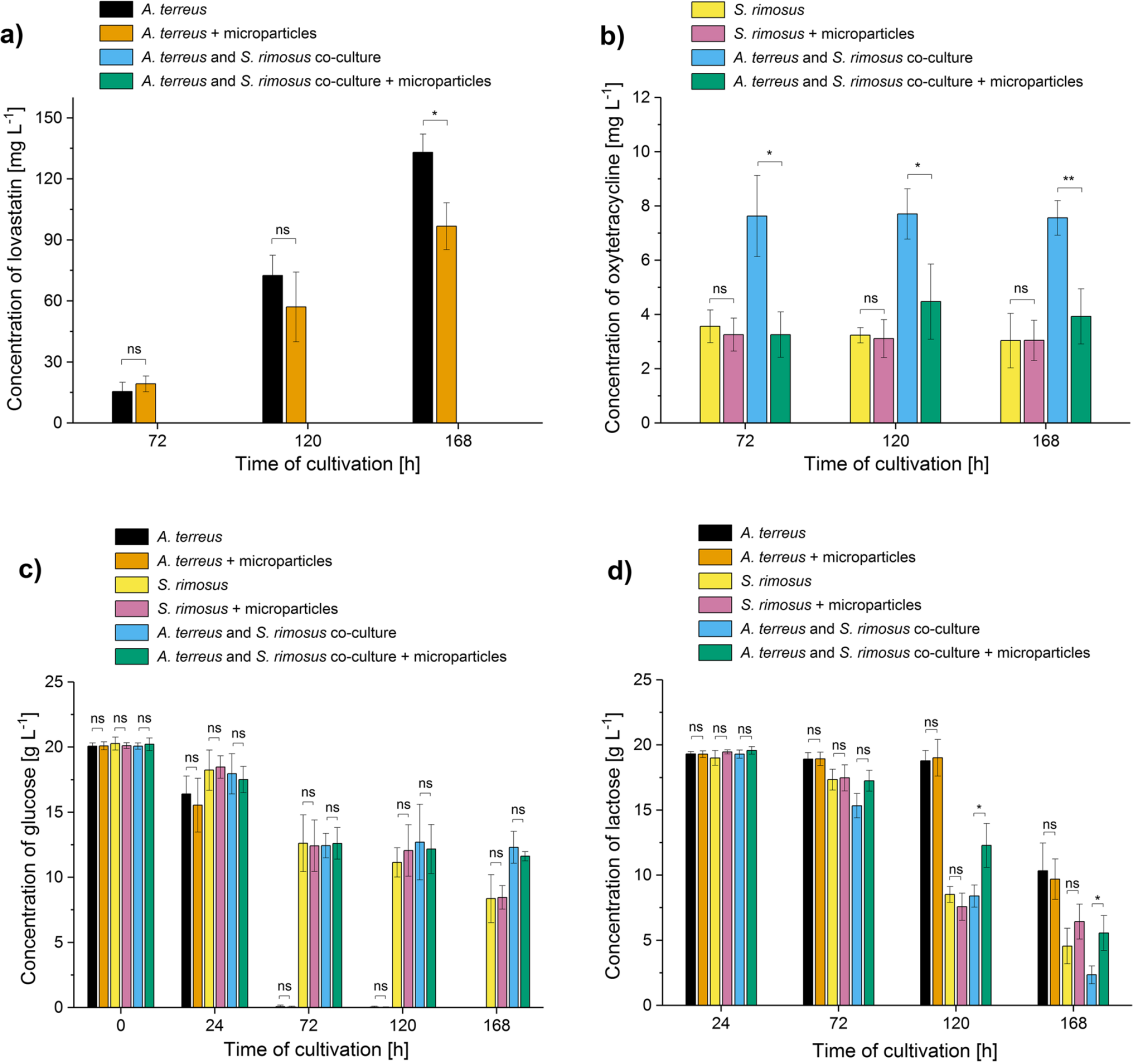
Influence of aluminum oxide (AO) on the titers of secondary metabolites and the levels of carbon substrates in the mono- and co-cultures of *Aspergillus terreus* and *Streptomyces rimosus*. Time courses of lovastatin (**a**), oxytetracycline (**b**), glucose (**c**), and lactose (**d**) levels in the *A. terreus* monoculture and/or *S. rimosus* monoculture and the “*A. terreus* vs. *S. rimosus*” co-culture with and without the addition of AO microparticles are shown. The concentration values are presented as “mean ± standard deviation” (*n* = 3). No traces of lovastatin or oxytetracycline were detected in 24 h of the cultivation run. The production of lovastatin was confirmed solely in *A. terreus* monocultures. The two-sample t-test (with a significance level of *α* = 0.05) was applied to verify if the results obtained for the AO variants differed significantly from their non-AO counterparts. *p ≤ 0.05, **p ≤ 0.01, ns, not significant

The production of lovastatin was recorded in *A. terreus* monocultures but not in the “*A. terreus* vs. *S. rimosus*” co-cultures (Fig. 6a). Importantly, the statistically significant increase of lovastatin titers in monocultures due to the presence of AO, which was observed earlier in the “*A. terreus* vs. *P. rubens*” study after 72 and 120 h of cultivation (Fig. 3a), was not recorded here (Fig. 6a). In the case of oxytetracycline (Fig. 6b), which was recorded both in the mono- and the co-cultivation, an interesting observation was made. Whereas the addition of AO failed to visibly affect the oxytetracycline levels reached in monocultures, it markedly impaired the production in co-cultures. While applying the approach of non-AO co-cultivation clearly elevated the titers of oxytetracycline compared to the AO- and non-AO monocultures, the addition of AO was responsible for reversing this positive effect and ultimately decreasing the antibiotic levels down to the values comparable with the ones recorded for the monocultures (Fig. 6b). Similarly as in the “*A. terreus* vs. *P. rubens*” case study, ( +)-geodin was detected in the broth at 120 and 168 h, but its presence was confirmed solely in the monocultures. Furthermore, no statistically significant differences in ( +)-geodin levels were revealed among the variants (Fig. S2a). Regarding the production of rimocidin, recorded in the *S. rimosus* monocultures and the co-cultures, the supplementation with AO did not affect the monoculture variants, but it did have a negative impact on the co-cultures (Fig. S2b).

Adding AO to the mono- or co-cultures did not lead to any marked changes with respect to glucose utilization (Fig. 6c). It was noted that the level of glucose was close to 0 after 72 h of *A. terreus* monocultivation regardless the presence of AO, whereas in the remaining variants, only approx. 50% of supplied glucose was catabolized at the end of the run (168 h). At this time, the concentration of residual glucose in co-cultures was higher than the one recorded for the monocultures (Fig. 6c). This stood in contrast with the results obtained earlier for the “*A. terreus* vs. *P. rubens*” co-cultures, in which the complete depletion of glucose was noted earlier than for the corresponding monocultures (Fig. 3c). As far as lactose was concerned, its consumption in the *S. rimosus*-involving variants was greater than in the *A. terreus* monocultures (Fig. 6d). It was also noticed that the addition of AO led to the delay of lactose uptake in the co-cultures (Fig. 6d).

### *A. terreus* versus *C. unicolor*

**Fig. 7:**
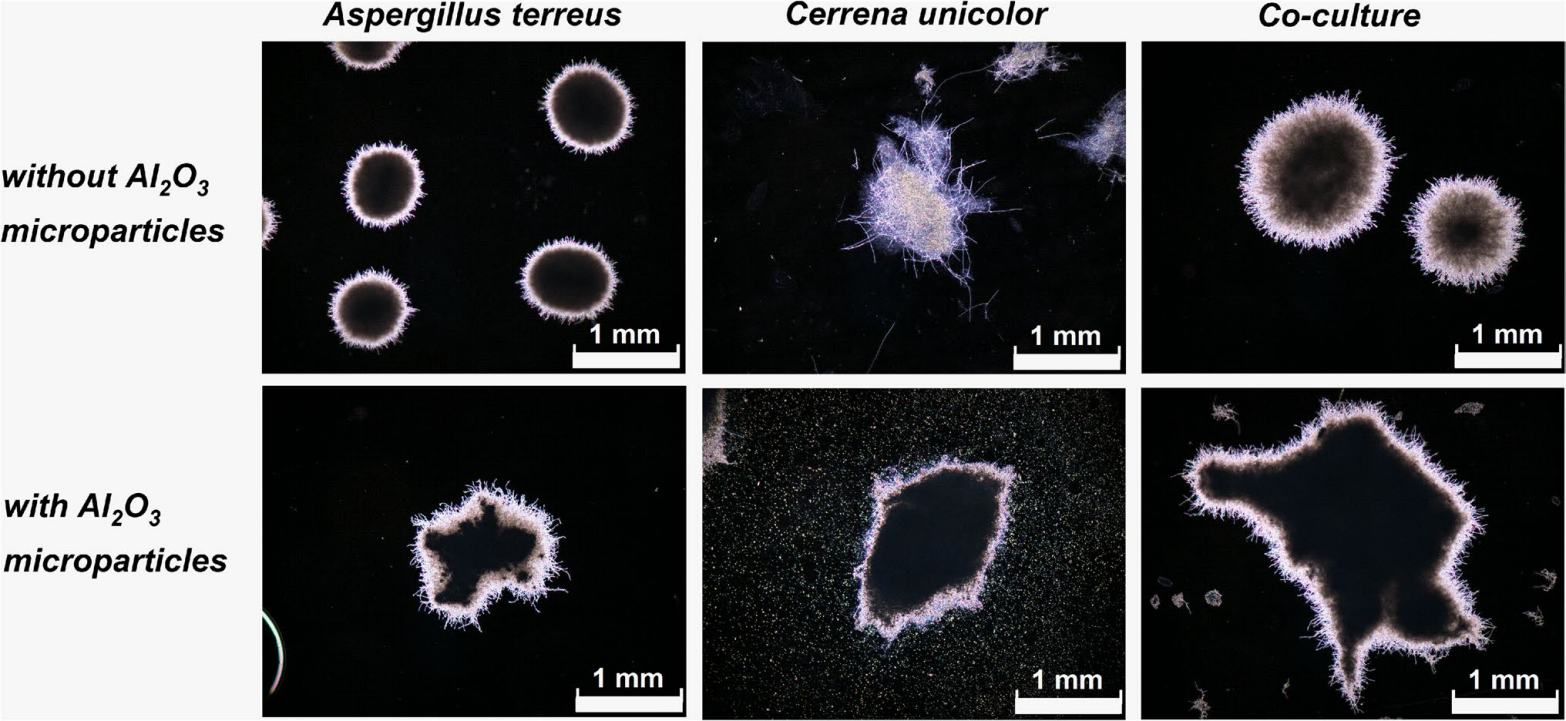
Influence of aluminum oxide (AO) on the morphology of *Aspergillus terreus* and *Cerrena unicolor* grown in mono- and co-cultures. Microscopic images of *A.* *terreus* monoculture, *C. unicolor* monoculture and the “*A. terreus* vs. *C. unicolor*” co-culture with and without the addition of AO microparticles are shown. The images were obtained after 24 h of cultivation. The presented examples illustrate the increase in pellet size due to the addition of AO in all tested variants. The medium used for the cultivation contained glucose (10 g L^−1^), yeast extract (2 g L^−1^), KH_2_PO_4_ (0.47 g L^−1^), L-asparagine (1.5 g L^−1^), thiamine (50 µg L^−1^), and salts. At least 45 objects were analyzed for each experimental variant, the selected examples are depicted here

**Fig. 8:**
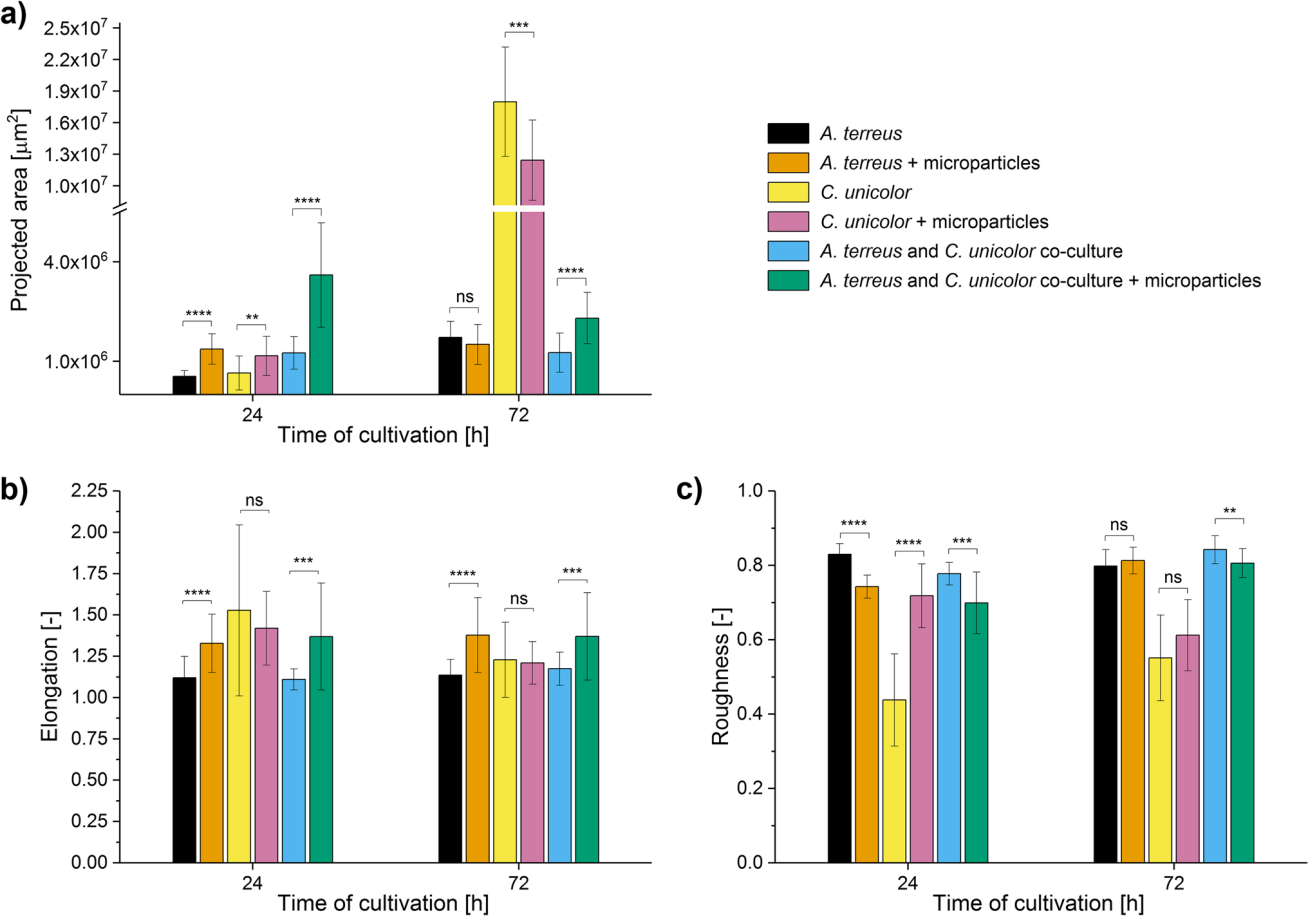
Influence of aluminum oxide (AO) on the values of morphological parameters determined for *Aspergillus terreus* and *Cerrena unicolor* grown in mono- and co-cultures. Results of quantitative morphological analysis of pellets developed in the *A. terreus* monoculture, *C. unicolor* monoculture, and the “*A. terreus* vs. *C. unicolor*” co-culture with and without the addition of AO microparticles are shown. The pellets of *C. unicolor* at 96 h became too large to conduct the microscopic observations (as in the work of Antecka et al. 2016b), so the morphological investigation in the “*A. terreus* vs. *C. unicolor*” case study was conducted until 72 h of the run. The values of projected area (**a**), elongation (**b**), and roughness (**c**) parameters are presented as the mean value ± standard deviation with the average number of analyzed objects (*n*) equal to 45. The two-sample *t*-test (with a significance level of *α* = 0.05) was applied to verify if the results obtained for the AO variants differed significantly from their non-AO counterparts. **p ≤ 0.01, ***p ≤ 0.001, ****p ≤ 0.0001, ns, not significant

The pellets of *C. unicolor* at 96 h became too large to conduct the microscopic observations (as in the work of Antecka et al. 2016b), so the morphological investigation in the “*A. terreus* vs. *C. unicolor*” case study was conducted until 72 h of the run. Already after 24 h of growth, the impact of AO was clearly visible, as the microparticles formed a compact and dense core within the developing pellets (Fig. 7). At 24 h, the mean projected area of all three tested variants, i.e., the monocultures of *A. terreus* and *C. unicolor* and the “*A. terreus* vs. *C. unicolor*” co-culture was increased due to AO supplementation. After 72 h, however, the AO-related increase of the mean projected area could still be observed only in the AO-containing co-cultures (Fig. 8a). Regarding the elongation parameter (Fig. 8b), it was noticed that the variability of values (indicated by error bars) recorded for the *A. terreus* monocultures and the co-cultures increased due to the addition of AO, while the opposite statement was true in the case of *C. unicolor* monocultures. At this point, it was also noticed that AO led to the increase in elongation in *A. terreus* monocultures and the co-cultures regardless the medium composition (this behavior was observed in all three investigated case studies, not only in the “*A. terreus* vs. *C. unicolor*” experiment). Finally, the statistically significant AO-related decrease and increase of roughness values recorded after 24 h for the monocultures of *A. terreus* and *C. unicolor*, respectively, could no longer be observed after 72 h (Fig. 8c). At this moment of the cultivation process, the pellets formed in the monocultures of *C. unicolor* stood out as the ones having the largest mean projected area (Fig. 8a) and, at the same time, the smallest mean roughness values (Fig. 8c) among the investigated variants.

**Fig. 9:**
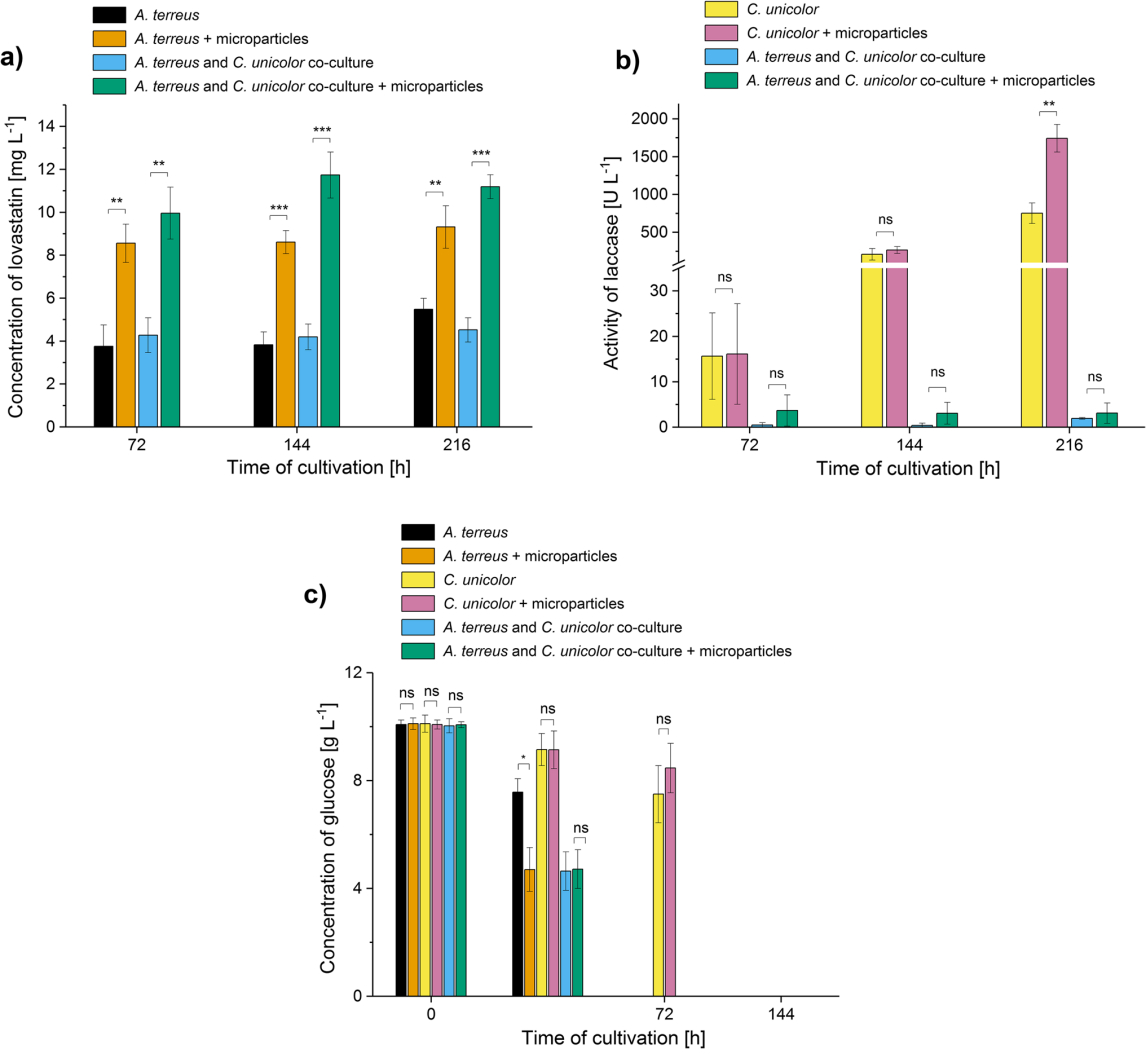
Influence of aluminum oxide (AO) on the titers of lovastatin and laccase and the levels of glucose in the mono- and co-cultures of *Aspergillus terreus* and *Cerrena unicolor*. Time courses of lovastatin (**a**), laccase (**b**), and glucose (**c**) levels in the *A. terreus* monoculture and/or *C. unicolor* monoculture and the “*A. terreus* + *C. unicolor*” co-culture with and without the addition of AO microparticles are shown. The concentration values are presented as “mean ± standard deviation” (*n* = 3). No traces of lovastatin or laccase activity were detected in 24 h of the cultivation run. As shown in (**c**), glucose was no longer detectable after 144 h of the run in all the tested variants. The cultivation was performed for 216 h as this time is required to achieve relatively high levels of laccase (Antecka 2016b). The two-sample *t*-test (with a significance level of *α* = 0.05) was applied to verify if the results obtained for the AO variants differed significantly from their non-AO counterparts. *p ≤ 0.05, **p ≤ 0.01, ***p ≤ 0.001, ns, not significant

The presence of AO led to the improvement of lovastatin production not only in the *A. terreus* monocultures but also in “*A. terreus* vs. *C. unicolor*” co-cultures (Fig. 9a). It was noticed that in the “*A. terreus* vs. *C. unicolor*” case study, all variants displayed lovastatin titers smaller than 12 mg L^−1^ (Fig. 9a), whereas in the case studies involving *P. rubens* (Fig. 3a) or *S. rimosus* (Fig. 6a), some of the tested variants exhibited lovastatin concentration values even greater than 120 mg L^−1^. While *S. rimosus* and *P. rubens*, the microbial species chosen to serve as partners of *A. terreus* in co-cultures, are generally referred to as the antibiotics (i.e., secondary metabolites) producers, *C. unicolor* is primarily described in the context of biosynthesizing and secreting laccases (i.e., enzymes). Although this study was meant to be focused on secondary metabolites, the activity of laccase was also analyzed here to get a comprehensive illustration of the AO influence on submerged co-cultures (Fig. 9b). According to the results, the addition of AO did lead to the enhancement of laccase production in monocultures, but this effect became visible in 216 h the run. In the co-cultures, however, AO did not cause the significant changes of laccase activity. Furthermore, the levels of laccase in co-cultures were very low compared to the results obtained for the corresponding monocultures (Fig. 9b). Finally, no traces of ( +)-geodin were found in the broth, what was not surprising considering the relatively low levels of lovastatin obtained here (Fig. 9a).

Unlike in the two preceding case studies, glucose was the only sugar included in the medium. After 24 h, the AO monoculture of *A. terreus* displayed lower glucose levels than its non-AO counterpart (Fig. 9c). By contrast, the consumption of glucose in the *C. unicolor* monocultures and in the “*A. terreus* vs. *C. unicolor*” co-cultures was not affected by AO. After 72 h, glucose was found exclusively in the *C. unicolor* monocultures, while after 144 h, no traces of this sugar were found in the tested sets (Fig. 9c).

## Discussion

The data gathered over the course of three case studies revealed the differences in morphological and biosynthetic responses among the monocultures and their co-culture counterparts. An example was noted in the “*A. terreus* vs. *P. rubens*” series of experiments, where the addition of AO influenced the production of lovastatin in a markedly different way in the investigated mono- and co-cultures. As far as the monocultivations of *A. terreus* were concerned, the mean levels of lovastatin in the 72 and 120 h of the run were visibly higher in the AO monocultures than in the non-AO variants. In the co-cultures, by contrast, the supplementation with AO led to the marked inhibition of lovastatin biosynthesis throughout the co-cultivation process (Fig. 3a). In the same experimental run, adding AO to the co-culture ultimately led to the 20-fold decrease of mean projected area of mycelial structures at 168 h, whereas in the *A. terreus* and *P. rubens* monocultures, the decrease at the same time point was not more than threefold (Fig. 2a). Another example was recorded in the final day of the “*A. terreus* vs. *S. rimosus*” run, where the inhibitory effects of microparticles on the production of oxytetracycline were recorded for the co-cultures but not for the corresponding *S. rimosus* monocultures (Fig. 6b).

The ± SD (standard deviation) error bars in Figs. 2, 5, and 8 reflected the variability of the morphological parameter values corresponding to the observed filamentous structures. For example, the projected area of pellets at a particular time varied within a certain range, but the mean value and the SD provided a good representation of the projected area of the entire population of filamentous objects present in the culture. Based on the previously published reports, one may expect the SD values of morphological parameters to be relatively large (Kowalska et al. 2018). It should be emphasized, however, that the employed analytical procedure assumes the detection and characterization of at least 45 filamentous objects that contribute to a particular mean and SD value (as detailed in “Materials and methods”).

It was demonstrated previously that the morphological changes in the MPEC can be achieved only if the microparticles are added to the medium at the early stage of cultivation, i.e., at the stage of spores. Introducing the microparticles at the later stages of growth, when the pellets are already formed, is known to be an ineffective approach (Driouch et al. 2010a; Gonciarz and Bizukojć 2014). Therefore, in MPEC, the microparticles are typically added to the medium together with spores (Antecka et al. 2016a) or, when the spores are not available, with the freshly harvested free hyphae that are used for inoculation (Antecka et al. 2016b). In the context of co-cultivation, the common approach of starting the submerged microbial co-cultures is to inoculate the sterile medium with precultures or to combine the precultures at a specific ratio. For example, in an important work of Onaka et al. (2011), the co-cultures (referred to as “combined cultures”) were started by inoculating sterile medium with the precultures of two bacterial species. An alternative approach was applied by Meschke et al. (2012) who used the spores instead of precultures to start the process of co-cultivation. In the present study, the latter method was applied and the spores served as the inoculum. The idea was not to combine the separately propagated mono-species precultures that represent the defined morphological characteristics, but rather to provide an environment in which the spores of both species are free to interact with AO. Ultimately, the co-agglomeration of spores of both species with AO can provide a core for the development of filamentous pellets. The outcomes of such multispecies interactions with mineral microparticles have never been addressed before.

Considering the production levels and morphological characteristics recorded here, the “winner and loser” scenario could be observed in the co-cultures, i.e., one of the strains being dominated by its partner. The “*A. terreus* vs. *S. rimosus*” co-cultivation was undoubtedly dominated by *S. rimosus*. This actinobacterium produced oxytetracycline (Fig. 6b) and rimocidin (Fig. S2b) in mono- and co-cultures, while its presence effectively prevented *A. terreus* from biosynthesizing lovastatin (Fig. 6a) and ( +)-geodin (Fig. S2a). This observation agreed with our recent study, which demonstrated the domination of *S. rimosus* over *A. terreus* unless a certain time advantage was provided for the fungus (Boruta et al. 2021). In the “*A. terreus* vs. *C. unicolor*” case, the morphological resemblance between the co-culture pellets and *A. terreus* monoculture pellets, as well as the fact that only the traces of laccase activity could be found in the co-cultures, suggested that *A. terreus* dominated its partner. In the “*A. terreus* vs. *P. rubens*” co-cultures, a lack of penicillin G and the morphological comparison indicated that the former fungus was a winner of the clash. However, it should be noted that *P. rubens* was not eradicated by *A. terreus*, since the secondary metabolite chrysogine was found in the co-cultures (Fig. S1b). During the preliminary experiments of a different study (Boruta et al. 2020), the co-cultures of *A. terreus* and *P. rubens* initiated with the use of spores did not reveal any traces of *P. rubens* metabolites due to the domination of *A. terreus* over *P. rubens*, what ultimately led to designing an alternative inoculation strategy. In the present work, the co-cultures were also initiated with the use of spores, so the presence of chrysogine in the co-cultivation broth was considered to be surprising. The discrepancy could be due to the differences in medium composition, specifically with respect to the presence of phenylacetic acid in the broth (this component was not used in the preliminary experiments of Boruta et al. 2020) or the concentration of yeast extract. It should be emphasized that choosing the “spores vs. spores” inoculation method in the present study was far from arbitrary, as described in “Materials and methods.” This approach is known to be effective in terms of allowing the microparticles to intertwine with the developing pellets and affect their production-related performance. It should be remembered that the “weaker” (dominated) species can still exert observable effects on the “stronger” (dominant) species even if its presence in the co-culture is not evidenced by metabolic profiles, as was recently shown for the “*A. terreus* vs. *S. rimosus*” microbial pair (Boruta et al. 2021). In other words, the less aggressive strain may act merely as a stimulatory factor that enhances the capabilities of the producing strain.

The domination of a given species over its microbial partner in co-culture can be evaluated on two levels, namely by comparing the repertoire of secondary metabolites and the morphological forms in mono- and co-cultivation variants. However, the results must be analyzed carefully by considering all available data rather than relying on a single morphological or biosynthetic characteristic. When it comes to assessing the dominant character of a given species in submerged co-culture, drawing the conclusions may not always be regarded as straightforward. The “*A. terreus* vs. *S. rimosus*” case study serves as a good example to illustrate the complexity associated with this issue. Upon the visual inspection of morphologies in mono- and co-cultures (Fig. 4), the comparison of projected area values (Fig. 5a) and the production of secondary metabolites (Fig. 6) the dominant role of *S. rimosus* was evident. The values of projected area recorded for the co-cultures were far from the ones observed in *A. terreus* monocultures and aligned much better with the values exhibited by *S. rimosus* monoculture. In addition, the impact of AO supplementation on *A. terreus* monoculture (i.e., smaller projected area of pellets) was not observed in the co-cultures (Fig. 5a). By contrast, if the values of roughness were considered, one may argue that the co-cultures resembled *A. terreus* monocultures in terms of the effects exerted by AO (i.e., the decrease of roughness value due to AO), which was not observed for *S. rimosus* monocultures (Fig. 5c). All in all, it can be stated that *S. rimosus* indeed dominated *A. terreus* in co-culture, but the presence of *A. terreus* still exerted some effects on the final outcomes of the co-cultivation process. It also showed that the assessment of domination of species in submerged co-culture needs to be based on strict and well-defined criteria to avoid confusion and misunderstandings. In the current study, which involved the microorganisms of distinct and easily distinguishable morphologies, the visual inspection of microscopic images (understood as a subjective and non-rigorous assessment of morphologies without consulting the values of parameters) seemed to be an effective method with regard to identifying the dominant species in the non-AO variants. It was not difficult to notice that the morphological forms visible in co-culture resembled the ones in the monoculture of the dominant species. For example, the “*A. terreus* vs. *P. rubens*” co-culture and the corresponding *A. terreus* monoculture were highly similar in terms of the developed morphological forms (Fig. 1), which was associated with the domination of *A. terreus* in co-culture. In the “*A. terreus* vs. *S. rimosus*” co-culture, the dominant role was taken by *S. rimosus*, which is why the morphologies recorded for the *S. rimosus* monoculture and the “*A. terreus* vs. *S. rimosus*” co-culture resembled each other very closely (Fig. 4). However, when the AO-influenced morphologies were compared, the visual identification of the dominant species was far more challenging. In the case of “*A. terreus* vs. *C. unicolor*” AO co-cultures, the subjective assessment of microscopic images was not found to be effective in terms of identifying the winner of the clash (Fig. 7), while the rigorous comparison of morphological parameters was seen as helpful (Fig. 8). Importantly, the domination of a given species in co-culture is not always noticeable solely by performing the visual inspection of microscopic images. If the considered co-cultivation system involved the microorganisms of similar (or even non-distinguishable) morphologies, the identification of the dominant species on the basis of morphological comparisons would not be feasible.

The correlation between the values of morphological parameters and the production level of lovastatin was dependent on medium composition and the mode of cultivation (i.e., mono- or co-culture). In the “*A. terreus* vs. *P. rubens*” case study the AO-induced decrease of projected area, the increase of elongation and decrease of roughness values due to the addition of AO (Fig. 2) led to the enhanced lovastatin formation in *A. terreus* monocultures but only in 72 and 120 h of the run (Fig. 3a). When *A. terreus* was co-cultured with *P. rubens*, the presence of AO also led to the decrease of projected area, increase of elongation, and decrease of roughness (Fig. 2), but these morphological changes were in this case associated with the decrease of lovastatin production (Fig. 3a). Hence, the correlation was dependent on whether the monoculture or the co-culture was analyzed. It demonstrates that the morphology-based conclusions formulated for the MPEC monocultures do not necessarily remain valid for the corresponding co-culture variants. This was also illustrated by the “*A. terreus* vs. *S. rimosus*” case study, where the production of lovastatin was blocked in co-cultures (Fig. 6a). It was not possible to predict such a behavior solely on the basis of morphological parameter values and a much broader perspective was required to understand the outcomes of co-cultivation. Since *S. rimosus* dominated *A. terreus* in co-cultures, the values of morphological parameters could not be of any use in terms of predicting the lovastatin titers. It demonstrates the shortcomings of the morphological parameter-based approach if the co-cultures, rather than monocultures, are under investigation.

What the three investigated case studies had in common was the participation of *A. terreus*. However, they were conducted with use of different media (but with the same type and concentration of AO). The (co-)cultivation media were chosen carefully for each pair of organisms based on literature (Antecka et al. 2016b; Boruta et al. 2020, 2021; Janusz et al. 2007) and tested with regard to supporting growth and the biosynthesis of target products by both participating organisms. While the stimulatory effect of microparticles on lovastatin production in monocultures was already described by Gonciarz and Bizukojć (2014) and then by Boruta and Bizukojc (2019), the dependency of the production-related and morphological outcomes of *A. terreus* cultivation on medium composition is demonstrated here for the first time. Briefly, according to the current results, the biosynthesis of lovastatin can be either stimulated or inhibited by the microparticles depending on the medium and the time of the process (Figs. 3a, 6a, and 9a). If the results of the present study were to be compared with the data presented by Gonciarz and Bizukojć (2014) and Boruta and Bizukojc (2019), it must be remembered that the studies differed in terms of medium composition and the duration of cultivation process. All in all, the present study confirmed the stimulatory effect of microparticles on lovastatin production by *A. terreus* and also demonstrated that the MPEC approach should not be perceived as a “magic bullet” that is universally effective regardless the bioprocess conditions. Furthermore, in the present work, the second metabolite of *A. terreus*, namely ( +)-geodin, was for the first time investigated in the context of MPEC. Inhibition of its production, noted here, can be regarded as greatly desired, as this metabolite is often considered as an unwanted by-product of lovastatin formation (Hasan et al. 2019). It should be noted that the effect of AO on the formation of ( +)-geodin was shown to be medium-dependent (compare Figs. S1a and S2a). In the “*A. terreus* vs. *P. rubens*” case study, the supplementation with AO led to the significant decrease of ( +)-geodin level in *A. terreus* monocultures (Fig. S1a), whereas in the “*A. terreus* vs. *S. rimosus*” experiment, this effect was not recorded (Fig. S2a). The differences in medium composition (e.g., the presence or absence of phenylacetic acid) were responsible for the medium-dependent effectiveness of AO, but further studies are required to uncover the details of this behavior. As the interactions between the microparticles, the growth medium and cultivated cells are intrinsically complex and occur at multiple levels, elucidating the molecular mechanisms associated with the medium-dependent impact of microparticles on the production of secondary metabolites is undoubtedly a challenging task (Laible et al. 2021).

Even though the present study did not involve any optimization efforts towards reaching the highest possible product titers, it perfectly illustrated the importance of setting the criteria for evaluating a medium composition. If lovastatin was taken as a target product, the candidate media could be assessed by considering the titer of lovastatin, the planned time of cultivation, and the level of ( +)-geodin, i.e., an unwanted by-product. If only the titer of lovastatin was taken into account here, performing the non-AO monoculture of *A. terreus* for 168 h, as in the “*A. terreus* vs. *P. rubens*” case study, would be the best option (Fig. 3a). However, if due to any reason, there was a necessity to shorten the total cultivation time from 168 to 120 h, adding AO to the medium that was used in the “*A. terreus* vs. *P. rubens*” case study and conducting the *A. terreus* monoculture for 120 h would be recommended (Fig. 3a), as at this time of the run, the AO variant showed higher lovastatin level than its non-AO counterpart (Fig. 3a). Finally, if the reduction of ( +)-geodin levels was seen as a key objective, performing the cultivation for 120 rather than 168 h should be considered, even at the cost of relatively lower lovastatin titers, since in *A. terreus* monocultures, the levels of ( +)-geodin were visibly higher at 168 than at 120 h (Figs. S1a and S2a).

The study demonstrated the importance of medium composition on the morphological outcomes of MPEC. For example, the values of projected area in the *A. terreus* monocultures did change in all the tested cases in response to the addition of AO; however, these changes were of distinct character depending on the medium composition. Specifically, in the case studies involving *P. rubens* or *S. rimosus*, the projected area of *A. terreus* pellets in monocultures was visibly smaller due to the presence of AO (Figs. 2a and 5a). This was definitely not the case in the experiments involving *C. unicolor* (Fig. 8a). The difference in terms of medium composition between the “*A. terreus* vs. *C. unicolor*” experiment and the remaining two cases was mainly associated with sugar and yeast extract content (as detailed in “Materials and methods”). According to the results, propagating *A. terreus* in a relatively rich medium (as in the experiments with *P. rubens* or *S. rimosus*) enabled AO to act as the pellet size-decreasing factor, whereas in a medium containing lower concentrations of sugar and yeast extract, this effect was not achieved. It indicates that the efforts of adjusting the concentration and type of microparticles in the MPEC optimization studies need to be accompanied by the development of medium that supports the effectiveness of microparticles towards reaching the desired morphological outcomes and, ultimately, satisfactory product titers.

The production of oxytetracycline by *S. rimosus* was recently shown to be enhanced by the addition of mineral microparticles (Kuhl et al. 2021). Here, AO failed to stimulate oxytetracycline biosynthesis (Fig. 6b). It should be noted that Kuhl et al. (2021) applied a different medium, a different type of microparticles (talc), and, finally, cultivated *S. rimosus* under the conditions that were not equivalent to the ones applied here. It proves once again that the application of microparticles to enhance product titers should not be perceived as a ready-to-use solution but rather as a promising basis for further process optimization efforts, as was mentioned above with regard to lovastatin formation. One of the key factors to consider is the concentration of microparticles. It was shown in the same work of Kuhl et al. (2021) that the presence of talc can lead either to the increase or decrease of oxytetracycline levels depending on its concentration in the medium. In many other studies focused on MPEC, the concentration of microparticles was found to be an important factor in the context of stimulating the production of metabolites and enzymes (Antecka et al. 2016b; Coban and Demirci 2016; Driouch et al. 2010a, 2012; Kaup et al. 2008; Gonciarz and Bizukojc 2014; Yatmaz et al. 2016). The mechanistic effects of microparticles on the formation of filamentous pellets were discussed by Driouch et al. (2010b). Briefly, microparticles interfere with spore agglomeration during the initial phase of pellet formation and the concentration of microparticles visibly affects the outcomes of this process.

The relatively low levels of lovastatin obtained in the “*A. terreus* vs. *C. unicolor*” case study were due to the medium composition. Based on our previous experience and the preliminary works regarding the growth rates and product formation in submerged mono- and co-cultures (not shown here), we decided to propagate the co-cultures in the medium tailored specifically towards *C. unicolor* development and laccase production (Antecka et al. 2016b). The rationale behind this choice was to provide a certain advantage to *C. unicolor*, a fungus exhibiting slower growth than the “more aggressive” *A. terreus*. Hence, the medium was far from optimal in the context of *A. terreus* growth and lovastatin formation, but the activity of *A. terreus* in terms of biomass development and metabolites production was still observed. In fact, even in the suboptimal medium, *A. terreus* managed to dominate *C. unicolor* in co-culture, as indicated by the laccase levels (Fig. 9b). At the same time, the presence of laccase activity in co-cultures (Fig. 9b) indicated that *C. unicolor* was not eliminated by *A. terreus*. To confirm that the laccase activity in co-cultures was due to *C. unicolor*, the samples drawn from *A. terreus* monocultures were also assayed, but no laccase activity was detected in them.

The study demonstrated that, within a given species, the microparticles can differently affect the production capabilities depending on the target metabolite. Such differences were observed, e.g., regarding the formation of lovastatin (Fig. 3a) and ( +)-geodin (Fig. S1a) in *A. terreus* monocultures. This is an important issue to consider over the course of optimization experiments, where, in principle, it is desired to promote the formation of target molecules while eliminating or at least lowering the levels of unwanted by-products, which are difficult and expensive to remove at the downstream processing stages.

The “cells versus cells” and “cells versus microparticles” interactions can be analyzed and discussed on multiple levels, e.g., metabolic, transcriptional, proteomic, morphological, and, importantly, with the focus of bioprocess performance. It is known that the microparticles alter the cellular metabolism of filamentous microorganisms by affecting the oxygen concentration profiles (i.e., oxygen availability to cells) inside the pellets (Gonciarz et al. 2016). If the microparticles are supplemented not to a conventional monoculture but to the co-culture, the picture gets far more complicated. In this case, the response of both microorganisms to the presence of microparticles opens the door for triggering new molecular interactions which are nonexistent in the non-microparticle co-cultivation variants. Considering the morphological evolution of filamentous organisms under submerged conditions, the species that dominates the co-cultures may “trap” the less aggressive species inside its pellets (Boruta et al. 2019), together with the co-agglomerating microparticles. Being influenced by the microparticles and the cells of other species bound together within the pellet core can be expected to have profound metabolic consequences on the producer strain. The response of the strain to microparticles alone or the accompanying species alone may thus differ from response elicited by the concerted action of both these factors.

Regarding the uptake of carbon substrates, it was noticed that in *A. terreus* monocultures, glucose was practically depleted at 72 h of the run regardless the medium (Figs. 3c, 6c, and 9c). This behavior was also seen in the “*A. terreus* vs. *P. rubens*” and “*A. terreus* vs. *C. unicolor*” co-cultures (Figs. 3c and 9c, respectively), in which *A. terreus* was a dominant microorganism. By contrast, in the “*A. terreus* vs. *S. rimosus*” co-culture, in which *S. rimosus* dominated, the glucose uptake pattern followed the one recorded for the *S. rimosus* monoculture and glucose was not seen to be depleted (Fig. 6c). The onset of lactose uptake in *A. terreus* monocultures depended on the medium composition. In the medium with an initial glucose concentration of 10 g L^−1^, lactose usage was recorded at 120 h (Fig. 3d), while in the medium containing glucose at the initial concentration of 20 g L^−1^, the level of lactose remained unchanged until 168 h of the run (Fig. 6d). In co-cultures, the time corresponding to the onset of lactose utilization followed the pattern recorded for the dominant species, i.e., *A. terreus* and *S. rimosus* in the “*A. terreus* vs*. P. rubens*” (Fig. 3d) and “*A. terreus* vs. *S. rimosus*” (Fig. 6d) case studies, respectively. It was also noticed that the influence of AO on the uptake of glucose in *A. terreus* monocultures during the first 24 h of growth depended on the initial concentration of this sugar in the medium. Specifically, in the medium containing 10 g L^−1^ of glucose, the AO-induced improvement of glucose utilization during the first 24 h was evident (Figs. 3c and 9c), whereas in the medium that was richer in glucose (20 g L^−1^), the difference between the AO and non-AO variant was not found to be significant (Fig. 6c).

Regarding the future research efforts related to MPEC, several possibilities are worth mentioning. Firstly, alternative methods of co-culture initiation can be assessed. For example, instead of directly inoculating the production medium with the spores accompanied by the microparticles, as was done here, one may follow the scheme of 2-stage cultivation, i.e., propagate the liquid precultures in the presence of microparticles and then use these precultures for inoculating the production medium. In such a case, the participating organisms would have a chance to develop individually prior to their confrontation. It also means that the microparticle-influenced morphologies would practically develop already at the preculture stage, unlike in the present study. Secondly, investigating the influence of microparticle concentration deserves further consideration. Thirdly, the current study purposedly focused on the industrially relevant species and their main metabolic products. The reasonable continuation would be to investigate the impact of microparticles on the production of secondary metabolites which are formed exclusively in co-cultures and, if possible, elucidate the molecular response of cells to microparticles in the presence of the accompanying species. Finally, moving towards the bioreactor-scale studies to explore the effects of mechanical stress on the morphology and secondary metabolites production would broaden the perspective on the topic.

To conclude, combining the submerged co-cultivation with MPEC was suggested and evaluated in the context of production-related performance and quantitative morphological characteristics of biotechnologically relevant filamentous species. Firstly, the study demonstrated that the effects exerted by mineral microparticles on the production levels in co-cultures and the corresponding monocultures can differ considerably, even if one of the co-cultivated species clearly dominates over its partner. Secondly, it was shown that the outcomes of MPEC are greatly dependent on medium composition and the duration of the cultivation process. For instance, the influence of AO on the production of lovastatin in *A. terreus* monocultures can change from stimulatory to inhibitory at a certain time point or remain stimulatory throughout the cultivation process, depending on the chosen medium. Finally, for the first time, the co-cultivation of *A. terreus* (representing *Ascomycetes*) with *C. unicolor* (*Basidiomycetes*) was investigated.

## Supplementary Information

Below is the link to the electronic supplementary material.

Supplementary file1 (PDF 274 KB)

## Data Availability

All data generated or analyzed during this study are included in this published article [and its supplementary information files].

## References

[CR1] Agustin MB, Carvalho DM, Lahtinen MH, Hilden K, Lundell T, Mikkonen KS (2021). Laccase as a tool in building advanced lignin-based materials. Chemsuschem.

[CR2] Antecka A, Bizukojc M, Ledakowicz S (2016). Modern morphological engineering techniques for improving productivity of filamentous fungi in submerged cultures. World J Microbiol Biotechnol.

[CR3] Antecka A, Blatkiewicz M, Bizukojć M, Ledakowicz S (2016). Morphology engineering of basidiomycetes for improved laccase biosynthesis. Biotechnol Lett.

[CR4] Antecka A, Blatkiewicz M, Boruta T, Górak A, Ledakowicz S (2019). Comparison of downstream processing methods in purification of highly active laccase. Bioprocess Biosyst Eng.

[CR5] Askenazi M, Driggers EM, Holtzman DA, Norman TC, Iverson S, Zimmer DP, Boers ME, Blomquist PR, Martinez EJ, Monreal AW, Feibelman TP, Mayorga ME, Maxon ME, Sykes K, Tobin JV, Cordero E, Salama SR, Trueheart J, Royer JC, Madden KT (2003). Integrating transcriptional and metabolite profiles to direct the engineering of lovastatin-producing fungal strains. Nat Biotechnol.

[CR6] Bertrand S, Bohni N, Schnee S, Schumpp O, Gindro K, Wolfender JL (2014). Metabolite induction via microorganism co-culture: a potential way to enhance chemical diversity for drug discovery. Biotechnol Adv.

[CR7] Bizukojc M, Pawlak M, Boruta T, Gonciarz J (2012). Effect of pH on biosynthesis of lovastatin and other secondary metabolites by *Aspergillus terreus* ATCC 20542. J Biotechnol.

[CR8] Blatkiewicz M, Antecka A, Boruta T, Górak A, Ledakowicz S (2018). Partitioning of laccases derived from *Cerrena unicolor* and *Pleurotus sapidus* in polyethylene glycol – phosphate aqueous two–phase systems. Process Biochem.

[CR9] Böl M, Schrinner K, Tesche S, Krull R (2021). Challenges of influencing cellular morphology by morphology engineering techniques and mechanical induced stress on filamentous pellet systems—A critical review. Eng Life Sci.

[CR10] Boruta T, Bizukojc M (2016). Induction of secondary metabolism of *Aspergillus terreus* ATCC 20542 in the batch bioreactor cultures. Appl Microbiol Biotechnol.

[CR11] Boruta T, Bizukojc M (2019). Application of aluminum oxide nanoparticles in *Aspergillus terreus* cultivations: evaluating the effects on lovastatin production and fungal morphology. Biomed Res Int.

[CR12] Boruta T, Milczarek I, Bizukojc M (2019). Evaluating the outcomes of submerged co-cultivation: production of lovastatin and other secondary metabolites by *Aspergillus terreus* in fungal co-cultures. Appl Microbiol Biotechnol.

[CR13] Boruta T, Marczyk A, Rychta K, Przydacz K, Bizukojc M (2020). Confrontation between *Penicillium rubens* and *Aspergillus terreus*: investigating the production of fungal secondary metabolites in submerged co-cultures. J Biosci Bioeng.

[CR14] Boruta T, Ścigaczewska A, Bizukojć M (2021). “Microbial wars” in a stirred tank bioreactor: Investigating the co-cultures of *Streptomyces rimosus* and *Aspergillus terreus*, filamentous microorganisms equipped with a rich arsenal of secondary metabolites. Front Bioeng Biotechnol.

[CR15] Coban HB, Demirci A (2016). Enhancement and modeling of microparticle-added *Rhizopus oryzae* lactic acid production. Bioprocess Biosyst Eng.

[CR16] Coban HB, Demirci A, Turhan I (2015). Microparticle-enhanced *Aspergillus ficuum* phytase production and evaluation of fungal morphology in submerged fermentation. Bioprocess Biosyst Eng.

[CR17] Coban HB, Demirci A, Turhan I (2015). Enhanced *Aspergillus ficuum* phytase production in fed-batch and continuous fermentations in the presence of talcum microparticles. Bioprocess Biosyst Eng.

[CR18] Driouch H, Roth A, Dersch P, Wittmann C (2010). Optimized bioprocess for production of fructofuranosidase by recombinant *Aspergillus niger*. Appl Microbiol Biotechnol.

[CR19] Driouch H, Sommer B, Wittmann C (2010). Morphology engineering of *Aspergillus niger* for improved enzyme production. Biotechnol Bioeng.

[CR20] Driouch H, Hänsch R, Wucherpfennig T, Krull R, Wittmann C (2012). Improved enzyme production by bio-pellets of *Aspergillus niger*: targeted morphology engineering using titanate microparticles. Biotechnol Bioeng.

[CR21] Etschmann MMW, Huth I, Walisko R, Schuster J, Krull R, Holtmann D, Wittmann C, Schrader J (2015). Improving 2-phenylethanol and 6-pentyl-α-pyrone production with fungi by microparticle-enhanced cultivation (AO). Yeast.

[CR22] Gonciarz J, Bizukojc M (2014). Adding talc microparticles to *Aspergillus terreus* ATCC 20542 preculture decreases fungal pellet size and improves lovastatin production. Eng Life Sci.

[CR23] Gonciarz J, Kowalska A, Bizukojc M (2016). Application of microparticle-enhanced cultivation to increase the access of oxygen to *Aspergillus terreus* ATCC 20542 mycelium and intensify lovastatin biosynthesis in batch and continuous fed-batch stirred tank bioreactors. Biochem Eng J.

[CR24] Hasan H, Abd Rahim MH, Campbell L, Carter D, Abbas A, Montoya A (2019). Improved lovastatin production by inhibiting (+)-geodin biosynthesis in *Aspergillus terreus*. N Biotechnol.

[CR25] Hasan H, Abd Rahim MH, Campbell L, Carter D, Abbas A, Montoya A (2021). Increasing lovastatin production by re-routing the precursors flow of *Aspergillus terreus* via metabolic engineering. Mol Biotechnol.

[CR26] Houbraken J, Frisvad JC, Samson RA (2011). Fleming’s penicillin producing strain is not *Penicillium chrysogenum* but *P.*
*rubens*. IMA Fungus.

[CR27] Janusz G, Rogalski J, Szczodrak J (2007). Increased production of laccase by *Cerrena unicolor* in submerged liquid cultures. World J Microbiol Biotechnol.

[CR28] Karahalil E, Coban HB, Turhan I (2019). A current approach to the control of filamentous fungal growth in media: microparticle enhanced cultivation technique. Crit Rev Biotechnol.

[CR29] Kaup BA, Ehrich K, Pescheck M, Schrader J (2008). Microparticle-enhanced cultivation of filamentous microorganisms: increased chloroperoxidase formation by *Caldariomyces fumago* as an example. Biotechnol Bioeng.

[CR30] Kim JH, Lee N, Hwang S, Kim W, Lee Y, Cho S, Palsson BO, Cho BK (2021). Discovery of novel secondary metabolites encoded in actinomycete genomes through coculture. J Ind Microbiol Biotechnol.

[CR31] Kowalska A, Boruta T, Bizukojć M (2018). Morphological evolution of various fungal species in the presence and absence of aluminum oxide microparticles: comparative and quantitative insights into microparticle-enhanced cultivation (AO). MicrobiologyOpen.

[CR32] Kuhl M, Rückert C, Gläser L, Beganovic S, Luzhetskyy A, Kalinowski J, Wittmann C (2021). Microparticles enhance the formation of seven major classes of natural products in native and metabolically engineered actinobacteria through accelerated morphological development. Biotechnol Bioeng.

[CR33] Laible AR, Dinius A, Schrader M, Krull R, Kwade A, Briesen H, Schmideder S (2021). Effects and interactions of metal oxides in microparticle-enhanced cultivation of filamentous microorganisms. Eng Life Sci (in Press).

[CR34] Luti KJK, Mavituna F (2011). Elicitation of *Streptomyces coelicolor* with *E. coli* in a bioreactor enhances undecylprodigiosin production. Biochem Eng J.

[CR35] Luti KJK, Mavituna F (2011). *Streptomyces coelicolor* increases the production of undecylprodigiosin when interacted with *Bacillus subtilis*. Biotechnol Lett.

[CR36] Meschke H, Walter S, Schrempf H (2012). Characterization and localization of prodiginines from *Streptomyces lividans* suppressing *Verticillium dahliae* in the absence or presence of *Arabidopsis thaliana*. Environ Microbiol.

[CR37] Meyer V, Cairns T, Barthel L, King R, Kunz P, Schmideder S, Müller H, Briesen H, Dinius A, Krull R (2021). Understanding and controlling filamentous growth of fungal cell factories: novel tools and opportunities for targeted morphology engineering. Fungal Biol Biotechnol.

[CR38] Michniewicz A, Ullrich R, Ledakowicz S, Hofrichter M (2006). The white-rot fungus *Cerrena unicolor* strain 137 produces two laccase isoforms with different physico-chemical and catalytic properties. Appl Microbiol Biotechnol.

[CR39] Mulder KCL, Mulinari F, Franco OL, Soares MSF, Magalhães BS, Parachin NS (2015). Lovastatin production: from molecular basis to industrial process optimization. Biotechnol Adv.

[CR40] Onaka H, Mori Y, Igarashi Y, Furumai T (2011). Mycolic acid-containing bacteria induce natural-product biosynthesis in *Streptomyces* species. Appl Environ Microbiol.

[CR41] Petković H, Cullum J, Hranueli D, Hunter IS, Perić-Concha N, Pigac J, Thamchaipenet A, Vujaklija D, Long PF (2006). Genetics of *Streptomyces rimosus*, the oxytetracycline producer. Microbiol Mol Biol Rev.

[CR42] Pohl C, Polli F, Schütze T, Viggiano A, Mózsik L, Jung S, de Vries M, Bovenberg RAL, Meyer V, Driessen AJM (2020). A *Penicillium rubens* platform strain for secondary metabolite production. Sci Rep.

[CR43] Sauer M, Porro D, Mattanovich D, Branduardi P (2008). Microbial production of organic acids: expanding the markets. Trends Biotechnol.

[CR44] Schäberle TF, Orland A, König GM (2014). Enhanced production of undecylprodigiosin in *Streptomyces coelicolor* by co-cultivation with the corallopyronin A-producing myxobacterium, *Corallococcus coralloides*. Biotechnol Lett.

[CR45] Singh R, Kumar M, Mittal A, Mehta PK (2016). Microbial enzymes: industrial progress in 21st century. 3 Biotech.

[CR46] Song Z, Ma Z, Bechthold A, Yu X (2020). Effects of addition of elicitors on rimocidin biosynthesis in *Streptomyces rimosus* M527. Appl Microbiol Biotechnol.

[CR47] Van Den Berg MA (2011). Impact of the *Penicillium chrysogenum* genome on industrial production of metabolites. Appl Microbiol Biotechnol.

[CR48] van der Meij A, Worsley SF, Hutchings MI, van Wezel GP (2017). Chemical ecology of antibiotic production by actinomycetes. FEMS Microbiol Rev.

[CR49] Veiter L, Rajamanickam V, Herwig C (2018). The filamentous fungal pellet—relationship between morphology and productivity. Appl Microbiol Biotechnol.

[CR50] Walisko R, Moench-Tegeder J, Blotenberg J, Wucherpfennig T, Krull R (2015). The taming of the shrew - controlling the morphology of filamentous eukaryotic and prokaryotic microorganisms. Adv Biochem Eng Biotechnol.

[CR51] Wösten HAB (2019). Filamentous fungi for the production of enzymes, chemicals and materials. Curr Opin Biotechnol.

[CR52] Wucherpfennig T, Kiep KA, Driouch H, Wittmann C, Krull R (2010). Morphology and rheology in filamentous cultivations. Adv Appl Microbiol.

[CR53] Yang J, Li W, Bun Ng T, Deng X, Lin J, Ye X (2017). Laccases: Production, expression regulation, and applications in pharmaceutical biodegradation. Front Microbiol.

[CR54] Yatmaz E, Karahalil E, Germec M, Ilgin M, Turhan İ (2016). Controlling filamentous fungi morphology with microparticles to enhanced β-mannanase production. Bioprocess Biosyst Eng.

[CR55] Zhao Y, Song Z, Ma Z, Bechthold A, Yu X (2019). Sequential improvement of rimocidin production in *Streptomyces rimosus* M527 by introduction of cumulative drug-resistance mutations. J Ind Microbiol Biotechnol.

[CR56] Zhuang L, Zhang H (2021). Utilizing cross-species co-cultures for discovery of novel natural products. Curr Opin Biotechnol.

[CR57] Zouboulis CC, Piquero-Martin J (2003). Update and future of systemic acne treatment. Dermatology.

