# Supplementary material for: Co-cultivation of filamentous microorganisms in the presence of aluminum oxide microparticles
Source: Appl Microbiol Biotechnol. 2022 Jul 30;106(17):5459–77. doi: 10.1007/s00253-022-12087-7 (PMC9418094; doi:10.1007/s00253-022-12087-7)
Supplement: Supplementary file 1 — Supplementary file1 (PDF 274 KB) [file 253_2022_12087_MOESM1_ESM.pdf]

Supplementary material

Applied Microbiology and Biotechnology

"Co-cultivation of filamentous microorganisms in the presence of aluminum oxide microparticles"

Tomasz Boruta*, Anna Antecka

Lodz University of Technology, Faculty of Process and Environmental Engineering, Department of Bioprocess Engineering, ul. Wolczanska 213, 93-005 Lodz, Poland

* Corresponding author. Phone: +48 42 631 39 77; fax: +48 42 636 56 63

E-mail address: tomasz.boruta@p.lodz.pl

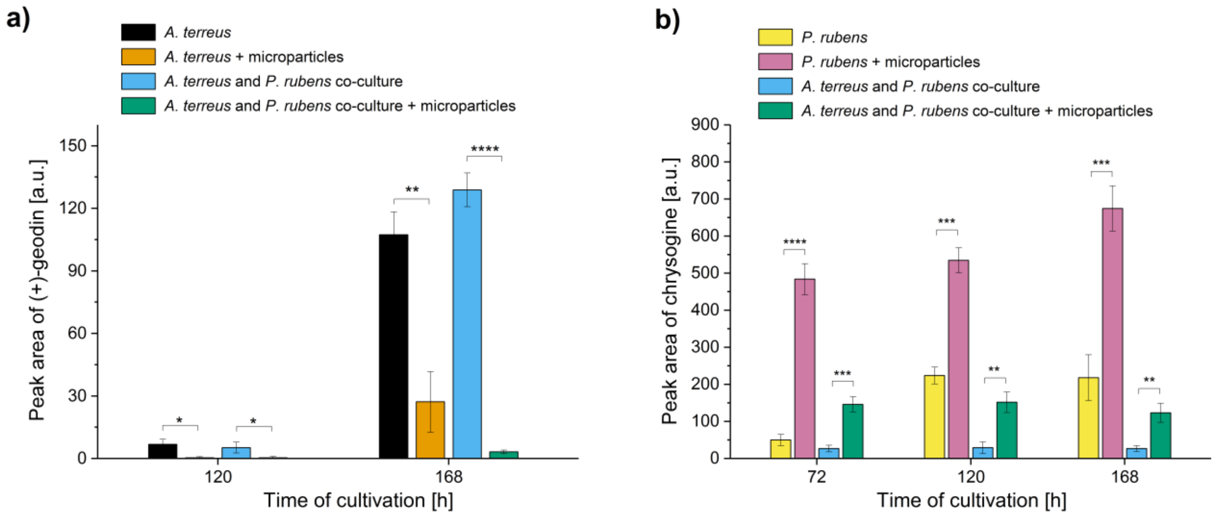

Figure S1 Influence of aluminum oxide (AO) on the titers of secondary metabolites in the mono- and co-cultures of *Aspergillus terreus* and *Penicillium rubens*. Time courses of (+)-geodin (a) and chrysogine (b) levels in the *A. terreus* monoculture or *P. rubens* monoculture and the "*A. terreus* vs. *P. rubens*" co-culture with and without the addition of AO microparticles are shown. The peak area values are presented as "mean ± standard deviation" ($n = 3$). The two-sample t-test (with a significance level of $\alpha = 0.05$) was applied to verify if the results obtained for the AO variants differed significantly from their non-AO counterparts. * $p \leq 0.05$, ** $p \leq 0.01$, *** $p \leq 0.001$, **** $p \leq 0.0001$, ns - not significant, a.u. - auxiliary units.

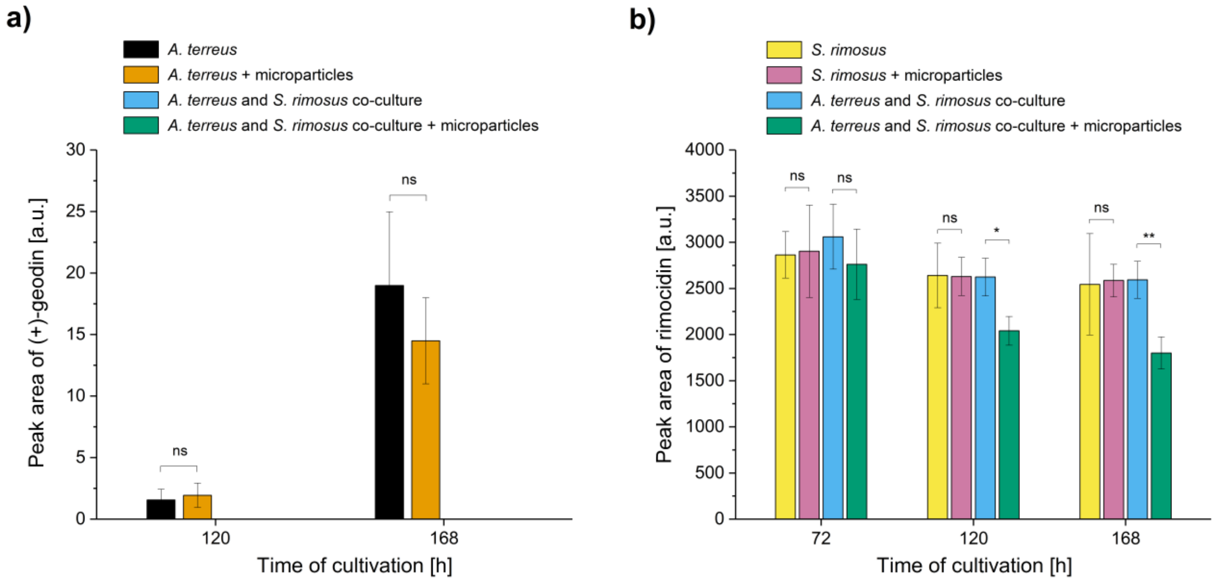

Figure S2 Influence of aluminum oxide (AO) on the titers of secondary metabolites in the mono- and co-cultures of *Aspergillus terreus* and *Streptomyces rimosus*. Time courses of (+)-geodin (a) and rimocidin (b) levels in the *A. terreus* monoculture or *S. rimosus* monoculture and the "*A. terreus* vs. *S. rimosus*" co-culture with and without the addition of AO microparticles are shown. The peak area values are presented as "mean ± standard deviation" ($n = 3$). The two-sample t-test (with a significance level of $\alpha = 0.05$) was applied to verify if the results obtained for the AO variants differed significantly from their non-AO counterparts. * $p \leq 0.05$, ** $p \leq 0.01$, ns - not significant, a.u. - auxiliary units.
